# Heterogenous bioluminescence patterns, cell viability, and biofilm formation of *Photobacterium leiognathi* strains exposed to ground microplastics

**DOI:** 10.3389/ftox.2024.1479549

**Published:** 2024-11-27

**Authors:** Rener De Jesus, Sameera Iqbal, Sunil Mundra, Ruwaya AlKendi

**Affiliations:** ^1^ Department of Biology, College of Science, United Arab Emirates University, Al Ain, United Arab Emirates; ^2^ Khalifa Center for Genetic Engineering and Biotechnology, United Arab Emirates University, Al Ain, United Arab Emirates

**Keywords:** plastic pollution, quorum sensing, luminous bacteria, hormesis, ecophysiology

## Abstract

Microplastics (MPs) have been detected in various aquatic environments and negatively affect organisms, including marine luminous bacteria. This study investigated the differences in bioluminescence patterns, cell viability, and biofilm formation of *Photobacterium leiognathi* strains (LB01 and LB09) when exposed to various concentrations of ground microplastics (GMPs; 0.25%, 0.50%, 1%, or 2% [w/v] per mL) at 22°C or 30°C for 3.1 days (75 h) and 7 days. The strains exhibited heterogenous responses, including variable bioluminescence patterns, cell viability, and biofilm formation, due to the GMPs having effects such as hormesis and bioluminescence quenching. Moreover, the bioluminescence and cell viability differed between the two strains, possibly involving distinct cellular mechanisms, suggesting that GMPs affect factors that influence quorum sensing. Furthermore, the biofilm formation of LB01 and LB09 was observed following exposure to GMPs. Both strains showed increased biofilm formation at higher GMP concentrations (1% and 2%) after 3.1 days at 30°C and 22°C. However, in the 7-day experiment, LB01 significantly (*p* < 0.05) increased biofilms at 22°C, while LB09 significantly (*p* < 0.05) produced biofilms at 30°C. These findings highlight the strain-specific responses of *Phb. leiognathi* to MP pollutants. Therefore, this study underscores the importance of evaluating MPs as environmental stressors on marine microorganisms and their role in the ecophysiological repercussions of plastic pollution in aquatic environments.

## 1 Introduction

The inexorable influx of plastics into the marine environment represents a profound ecological challenge, with microplastics (MPs) and nanoplastics emerging as pervasive contaminants (Alimi et al., 2018; Zhang and Xu, 2020). MPs, typically measuring <5 mm (<0.2 inches), are characterized by their small size, elasticity, and ability to permeate diverse environments, including aquatic ecosystems (Andrady, 2017; Mattson et al., 2018; Boyle and Örmeci, 2020). The extensive distribution of these plastic particles poses significant risks to aquatic life. Aquatic organisms, from microscopic zooplankton to large aquatic mammals, can ingest MPs that can cause adverse effects (Lusher et al., 2015; Rummel et al., 2016). In particular, the leaching of adsorbed or inherent toxic substances from ingested plastic particles (such as phthalates, bisphenol A, and persistent organic pollutants) can disrupt their endocrine and reproductive systems. These toxins can be transferred up the food chain, thereby posing risks to predator species and human health (Gallo et al., 2018). These risks extend not only to the organisms that form the backbone of aquatic food webs but also to marine microorganisms, including luminous bacteria (LB).

LB are fundamental to the maintenance of a marine ecosystem’s integrity, from nutrient recycling (Thompson and Polz, 2006) to the health of higher trophic organisms, which involves symbiosis (Stabb et al., 2008). LB are involved in nutrient turnover, breaking down organic materials and converting them into molecules for use by other marine organisms. Moreover, LB often engage in symbiotic interactions with fish and invertebrates, providing them with benefits such as camouflage, attraction of prey, or deterrence of predators due to their light-emitting capabilities. Examples of LB are species belonging to the genera of *Aliivibrio* (such as *Aliivibrio fisheri*, and *Aliivibrio salmonicida*), *Vibrio* (such as *Vibrio harveyi*), and *Photobacterium* (such as *Phb. phosphoreum*, and *Phb. leiognathi*). Many LB are equipped with a sophisticated cell-to-cell communication system known as quorum sensing (QS) system, which regulates numerous physiological responses including bioluminescence (Chong et al., 2013; Wang et al., 2022) and biofilm formation (Hammer and Bassler, 2003).

QS is mediated by the production and detection of signaling molecules, which enable the bacterial population to collectively coordinate gene expression and effectively “decide” when to activate certain genes based on the density of their population (Anetzberger et al., 2009; Lupp et al., 2003). This mechanism also influences cell viability in several ways, including by affecting virulence factor production, resource allocation, stress responses, and biofilm formation. The latter is particularly significant, with QS triggering a cascade of genetic and biochemical events that lead to the establishment, development, and maturation of biofilms, which are critical for bacterial survival and adaptation in various environments (Subramani and Jayaprakashvel, 2019). Therefore, this regulatory system enables bacteria within biofilms to optimize resource use, enhance defense mechanisms, and synchronize activities, such as dispersal or virulence, significantly impacting their interactions with hosts and the environment (Subramani and Jayaprakashvel, 2019; Warrier et al., 2021).

Environmental pollutants, including MPs and heavy metals, have been shown to affect QS in marine bacteria, with potential implications for their survival and ecological functions. For example, a range of compounds, including MP beads, Cu^2+^, Gd^3+^, and nanoAg, have been shown to affect QS in *A. fisheri* at non-toxic concentrations (Gagné, 2017). Although the changes were more modest, the study found that exposure to these compounds disrupted bioluminescence and altered the QS between *A. fisheri* cells. This is particularly noteworthy as it provides the first evidence that MPs and other toxic chemicals can disrupt bacterial QS. In addition, previous studies have reported that MPs acted as substrates for microbial communities that allowed biofilm communities to form and thrive (Tu et al., 2020; Valentin et al., 2016). In the present study, the impact of ground MPs (GMPs) on *Phb. leiognathi* was investigated, with a focus on three critical aspects: bioluminescence, cell viability, and biofilm formation. *Phb. leiognathi* is a marine LB that thrives in symbiosis with certain marine organisms (e.g., fish and squids), and is also free-living in the ocean (Thirukumar et al., 2022; Naguit et al., 2014; Yaser et al., 2014). The presence of MPs in aquatic ecosystems has raised concerns about potential ecophysiological effects. By observing changes in bioluminescence, the present study assessed the effects of GMPs on *Phb. leiognathi* QS. Additionally, using MTT assays provided insights into the effects of GMPs on bacterial viability. A post-exposure recovery experiment was conducted to evaluate whether *Phb. leiognathi* could achieve the same state as the unexposed group, using bioluminescence response as an indicator, after GMP exposure. Finally, examining the biofilm formation by *Phb. leiognathi* helps us to understand the implications of plastic pollution for bacterial colonization. Hence, this study contributes to the understanding of how plastic particles as environmental stressors affect LB and their potential ecophysiological roles in aquatic environments.

## 2 Materials and methods

### 2.1 LB isolation and purification

Freshly caught squids were bought from a local wet market in Al Ain, United Arab Emirates. The squids were placed in an ice box and transported immediately to the laboratory. The squids were dissected, and the ink sac was identified. The ink sac was gently opened using a sterile scalpel and the ink was collected by absorption using a sterile cotton swab and aseptically transferred into a test tube containing 0.85% saline solution. The solution was then serially diluted up to 10^–4^ and 0.1 mL was spread plated onto sea water agar (SWA; HiMedia^®^) plates and incubated at 28°C for 48 h. The spread plating was conducted in duplicates. After incubation, each plate was visually observed in the dark for LB colonies. The plates without LB colonies were further incubated for another 24 h. To obtain pure isolates, an LB colony from each plate was picked using an inoculating needle, streaked onto a new SWA plate, and observed after incubation at 28°C for 48 h. The pure LB isolates were then subjected to bioluminescence screening.

### 2.2 Bioluminescence screening

To determine the bioluminescence intensity and select the most suitable isolates for further study, 14 LB isolates were subjected to bioluminescence screening (Bao et al., 2023). The positive control was *Escherichia coli* DH108 transformed with plasmid pJE202 expressing the Lux operon genes from *Vibrio fisheri*. Overnight cultures of the LB isolates were incubated on SWA plates at 28°C for 48 h. *E. coli* DH108 was incubated on a plate containing Luria–Bertani agar with ampicillin at 35°C for 48 h. After incubation, colonies were picked from the plates and transferred to a test tube containing 0.85% saline solution. The cell density of each suspension was determined with reference to the 0.5 McFarland standard (∼1.5 × 10^8^ CFU/mL). A loopful (HiMedia^®^ Hi-FlexiLoop 2; 2.0 mm in diameter, calibrated to 0.005 mL) of each suspension was inoculated in tubes containing 10 mL artificial sea water broth (HiMedia^®^) with 0.25% [w/v] yeast extract (HiMedia^®^) and 0.5% [w/v] tryptone type-1 (HiMedia^®^) (ASW-YE-T) and incubated at 28°C while shaking (120 rpm) in a shaking incubator (Bioevopeak Co., Ltd., shaking incubator) for 48 h. For *E. coli* DH108, Luria–Bertani broth with ampicillin was used. After incubation, 200 µL of inoculum from each culture was transferred to a well of a microtiter plate (black, flat bottom, 96 wells, Costar^®^), with nine replicates. The microtiter plate was covered with foil and left to stand for 10 min before reading. The bioluminescence intensity was measured using a GloMax^®^ Discover Microplate Reader (Promega Corporation, Madison, WI, USA) with a 10-s integration period prior to reading. Bioluminescence is reported as specific bioluminescence (SB), which is the log-transformed values relative light unit (RLU). As a result, two LB strains were selected: LB01 and LB09. Stock cultures of these bacteria were maintained in cryovials containing 20% [w/v] NaCl–trypticase soy broth with 20% [v/v] glycerol and stored at − 20°C for further study.

### 2.3 Identification of LB isolates

#### 2.3.1 DNA extraction

DNA materials were extracted from the two LB strains using a G-spin™ genomic DNA extraction kit (iNtRON Biotechnology Inc., South Korea) following the manufacturer’s protocol. Briefly, 24-h old cultures were prepared and 1 mL aliquot was transferred into a microcentrifuge tube to collect cells by centrifugation (15,500 × *g* for 1 min). The supernatant was discarded and 300 µL of buffer solution was added. The tubes were vortexed and incubated in a heat block at 65°C for 15 min with gentle invert mixing every 5 min. Next, 250 µL of binding buffer was added and gently vortexed. Thereafter, the cell lysates (∼550 µL) were loaded into spin columns, centrifuged (15,500 × *g* for 1 min), and washed twice with 500 µL of washing buffer. The columns were then placed in a new microcentrifuge tube and 50 µL of elution buffer was added directly onto the membrane of each column. The tubes were incubated at 25°C for 1 min and then centrifuged (15,500 × g for 1 min). Finally, the DNA concentrations were measured using a NanoDrop™ 2000/2000c spectrophotometer (Thermo Fisher Scientific Inc., MA, USA).

#### 2.3.2 PCR amplification, purification, and gel electrophoresis

The extracted DNA was amplified using a PCR amplification kit (TaKaRa Bio Inc., Japan). The primers 27f (5′-AGA​GTT​TGA​TCC​TGG​CTC​AG-3′) and 1492r (5′-CTA​CGG​CTA​CCT​TGT​TAC​GA-3′) were purchased from Gene Link™, Inc. The PCR mixtures were prepared by combining 38.75 µL of sterile nuclease-free water, 2.5 µL of 10× PCR buffer, 2.5 µL of MgCl_2_, 4 µL of dNTP mixture, 0.5 µL of each primer, 1 µL of DNA sample, and 0.25 µL of Taq DNA Polymerase (TaKaRa). Touchdown PCR was performed using a T-100™ thermal cycler (Bio-Rad Laboratories Inc., Hercules, CA, USA) under the following conditions: initial denaturation at 95°C for 3 min; 29 cycles of denaturation at 95°C for 30 s, annealing at 68°C for 30 s, and extension at 72°C for 30 s; then another 29 cycles of denaturation at 95°C for 30 s, annealing at 60°C for 30 s, and extension at 72°C for 30 s; termination at 72°C for 5 min; and storage at 4°C until use. The PCR amplicons were purified using a MEGAquick-spin™ plus Total Fragment DNA Purification Kit (iNtRON Biotechnology Inc., South Korea). Next, 10 µL of the PCR amplicons was transferred to a microcentrifuge tube and 250 µL of lysis buffer was added. The mixture was then loaded into a column in a collection tube and centrifugation (at 11,000 × g for 30 s). Next, 750 µL of washing buffer was added to the column and centrifuged (at 11,000 × g for 30 s). To dry the column membrane, centrifugation at full speed (18,000 × g) for 3 min was conducted. Thereafter, the column was placed into a new microcentrifuge tube and 40 µL of elution buffer was added to the membrane center and left to stand for 1 min. Finally, centrifugation at full speed for 1 min was conducted to elute the DNA. The purified PCR amplicons were subjected to gel electrophoresis to check the quality and quantity. Next, 8 µL of each purified PCR product with 2 µL of loading dye was loaded onto 1% agarose gel well and run in 1X Tris–acetate–EDTA (TAE) buffer at 100 V for 45 min. A 1-kb molecular DNA ladder (New England Biolabs^®^, Beverly, MA, USA) was used. The gel was viewed under UV light using a Gel Doc™ EZ Imager with Image Lab™ software version 5.0 (Bio-Rad Laboratories Inc., Hercules, CA, USA).

#### 2.3.3 DNA sequencing

The purified PCR amplicons were subjected to Sanger sequencing and fragment analysis by capillary electrophoresis using a 3,500 Genetic Analyzer (Applied Biosystems, Thermo Fisher Scientific). The obtained sequences were checked and cleaned using 4peaks version 1.8 (www.nucleobytes.com). The cleaned sequences were run against the 16S ribosomal RNA sequence database for Bacteria and Archaea available from GenBank^®^ (https://www/ncbi.nlm.nih.gov) using Basic Local Alignment Search Tool (BLAST; National Center for Biotechnology Information, Bethesda, MD, USA). LB01 was identified as *Phb. leiognathi* subsp. *mandapamensis*, while LB09 was identified as *Phb. leiognathi*.

### 2.4 Preparation of ground microplastics (GMPs)

Microbeads, which made of polyethylene with inorganic metal pigments, extracted from cosmetic products by Habib et al. (2020) were used in this study. First, 10 g of microbeads were rinsed using type 1 ultrapure water to remove unwanted particulates, submerged in 70% ethanol for 30 min, washed with deionized water, and dried at 45°C in a hot air oven (Daihan Scientific Co., South Korea). The beads were ground using a sterile pestle and mortar and then exposed to UV light overnight. A small amount (0.01 g) of the GMPs were observed under a stereomicroscope (Leica Zoom 2000). The size of GMPs was measured using the ImageJ software (https://imagej.net/ij/) and the size distribution was determined (Supplementary Figure S1). To ensure asepsis, 0.1 g of GMPs was transferred into several tubes containing trypticase soy broth and incubated at 35°C for 48 h. After incubation, no tube showed turbidity.

### 2.5 Growth kinetics and bioluminescence profiles of *Phb. leiognathi* strains

The *Phb. leiognathi* strains in stock cultures were revived in tubes containing ASW-YE-T broth and incubated at 28°C for 48 h. Revived bacterial cells were collected by repeated centrifugation (6,000 × g for 10 min) and resuspended in phosphate-buffered solution (PBS, pH 7.4). The optical density at 600 nm (OD_600_) was determined and adjusted to 0.5–0.8 (Swift et al., 1993; Ahmad et al., 2012; Muneeswaran et al., 2021). To initially determine the optimal temperature for peak bioluminescence, an experiment was conducted across a temperature range of 15°C–35°C. After 48 h of incubation, bioluminescence intensity was measured at each temperature, revealing that the highest levels were observed at 22°C and 30°C. The said temperatures were selected for this study to characterize growth kinetics and bioluminescence of *Phb. leiognathi* strains.

Freshly revived bacterial cells were collected, and new suspensions were prepared. Subsequently, 3 µL of the suspension was inoculated into tubes containing 15 mL ASW-YE-T broth and incubated at 22°C or 30°C while shaking (120 rpm). The bacterial growth was monitored over time (until it reached the death phase) by transferring 200 µL to a microtiter plate well and measuring the OD_600_ using a GloMax^®^ Discover Microplate Reader (Promega Corporation, Madison, WI, USA).

### 2.6 GMP exposure experiment

To investigate the effects of GMPs on *Phb. leiognathi* strains, fresh cultures of the strains in the exponential growth phase were prepared. An aliquot was transferred into a microcentrifuge tube and the cells were harvested by centrifugation (6,000 × g for 10 min). The supernatant was carefully discarded, and the pellet was washed twice with PBS (pH 7.4) and then centrifuged again (6,000 × g for 10 min). After washing twice with PBS (pH 7.4), the supernatant was carefully discarded, ensuring the pellet remained undisturbed. The OD_600_ was adjusted to 0.5–0.8 and the bacterial suspension was immediately used for the GMP exposure experiment. Various GMP concentrations (0.25%, 0.50%, 1.00%, and 2.00% [w/v] per mL) were added to tubes containing 15 mL of ASW-YE-T broth. Control tubes contained ASW-YE-T broth without GMPs. Next, 10 µL of the bacterial suspension was transferred into each tube. The tubes were then incubated at 22°C or 30°C while shaking (120 rpm) in a shaking incubator (Bioevopeak Co., Ltd.). The bioluminescence responses of the bacteria to different GMP concentrations and temperatures were monitored over two timeframes: (1) every 5 h for 3.1 days (75 h) to assess immediate effects, and (2) every 24 h for 7 days to assess long-term effects. To prevent nutrient depletion, double-strength ASW-YE-T broth was added in the 7-day experiment. To assess the bioluminescence, 200 µL (n = 3) from each tube was transferred to each well of a black microtiter plate. The plate was covered with foil and left undisturbed for 10 min. SB was then measured as described in the Bioluminescence Screening section. This experiment was conducted three times on separate occasions, with triplicate wells (n = 9).

### 2.7 Post-exposure recovery experiment

This experiment was conducted to determine whether *Phb. leiognathi* strains previously exposed to GMPs for 3.1 days and 7 days can recuperate based on their bioluminescence intensities. A 1-mL aliquot of the bacterial culture was carefully pipetted from the tubes in 3.1-day and 7-day GMP exposure experiment, transferred into a microcentrifuge tube, and centrifuged (6,000 × g for 10 min). Next, the supernatant was carefully discarded, the pellet was washed three times with PBS (pH 7.4), and then resuspended in PBS (pH 7.4). The OD_600_ was measured, adjusted to 0.5–0.8, and 10 µL of each prepared bacterial suspension was transferred into tubes containing 15 mL ASW-YE-T broth. The tubes were then incubated for 35 h at 22°C or 30°C while shaking (120 rpm). The bioluminescence responses of the strains were monitored every 5 h for 35 h. Then, 200 µL (n = 3) from each tube was transferred to the wells of a black microtiter plate. The plate was covered with foil and left undisturbed for 10 min. Next, the SB was measured as described in the Bioluminescence Screening section. This experiment was conducted three times on separate occasions, with triplicate wells (n = 9).

### 2.8 MTT assay

The cell viability of *Phb. leiognathi* strains after exposure to varying GMP concentrations were assessed using MTT (3-(4,5-dimethylthiazol-2-yl)-2,5-diphenyltetrazolium bromide) assays with modifications (Wang et al., 2010). To prepare the MTT solution, 50 mg of MTT powder (bioWORLD, GeneLinx International, Inc. USA) was dissolved in 10 mL deionized water while stirring at 37°C. The solution was stored in microcentrifuge tubes at − 20°C until use. 3 μL of each bacterial suspension was inoculated in a well of a microtiter plate (clear, flat bottom, 96 wells, Costar^®^) containing 100 µL of ASW broth. The plate was incubated at 22°C or 30°C for 8 h. Next, 10 µL of MTT solution was added using a multi-channel pipette (Eppendorf Research^®^) and left to stand for 20 min. Thereafter, the broth was removed by gentle pipetting and 100 µL of dimethyl sulfoxide (purity >99%; Merck, Darmstadt, Germany) was added. The OD_560_ was measured using a microtiter plate reader after shaking the plate for 10 s. Each OD value was corrected based on the blank (medium without cells), and the mean corrected OD values were then calculated. This experiment was conducted with replicates (n = 12).

### 2.9 Crystal violet biofilm formation assay

The biofilm formation of *Phb. leiognathi* strains after GMP was assessed using the method (De Jesus and Dedeles, 2020) with modifications. To initiate biofilm formation, 1% (w/v) squid’s ink solution was used to coat the wells of a microtiter plate (clear, flat bottom, 96 wells, Costar^®^). The ink was collected from a squid’s ink sac using a sterile syringe and added to sterile deionized water. The ink solution was heated at 55°C for 10 min and then 100 µL was transferred to the wells and the microtiter plate was placed in an incubator overnight. Next, the contents were removed from the wells and 100 µL of double-strength ASW-YE-T broth was added to the wells. The wells were then inoculated with 3 µL of bacterial suspension and incubated at 22°C or 30°C while shaking (150 rpm) for 48 h. After incubation, the broth was removed by gentle pipetting, replenished with freshly prepared ASW-YE-T to support the biofilm formation, and incubated again at the previously mentioned temperatures for 48 h. The growth in microtiter plate wells was assessed based on the OD_600_ using the GloMax^®^ Discover Microplate Reader. Using a multi-channel pipette (Eppendorf Research^®^), the broth of each well was removed, and the wells were gently washed once with 200 µL of sterile PBS (pH 7.2), and then air-dried for 20 min. The attached biofilms in the wells were stained with 130 µL of 1% (v/v) crystal violet solution (HiMedia^®^) for 5 min and washed three times with 200 µL of sterile distilled water. The stained biofilms in the wells were solubilized with 130 µL of absolute ethanol (Carlo Erba, Milan, Italy) and the OD_560_ was assessed. The results were expressed as specific biofilm formation (SBF), calculated as *(A* – *B)/C,* where *A* is the OD_560_ of the stained biofilms, *B* is the OD_560_ of the stained blank control wells (to eliminate non-specific or abiotic OD values), and *C* is the OD_600_ of bacterial growth in ASW-YE-T broth. The assay was performed in replicates (n = 9).

### 2.10 Data analysis

The bioluminescence intensities were normalized (min-max scaling; 0–1) to reduce potential errors. The differences in mean OD values in MTT assays and the mean SBF in biofilm formation assays (control groups vs. exposed groups) were examined using two-way ANOVA, followed by Dunnett’s *post hoc* analysis in Graphpad Prism 10 (version 10.2.3). *p* < 0.05 was accepted as statistically significant, indicating reliable differences between control groups and exposed groups.

## 3 Results

### 3.1 Bioluminescence and growth dynamics of *Phb. leiognathi* strains

**FIGURE 1:**
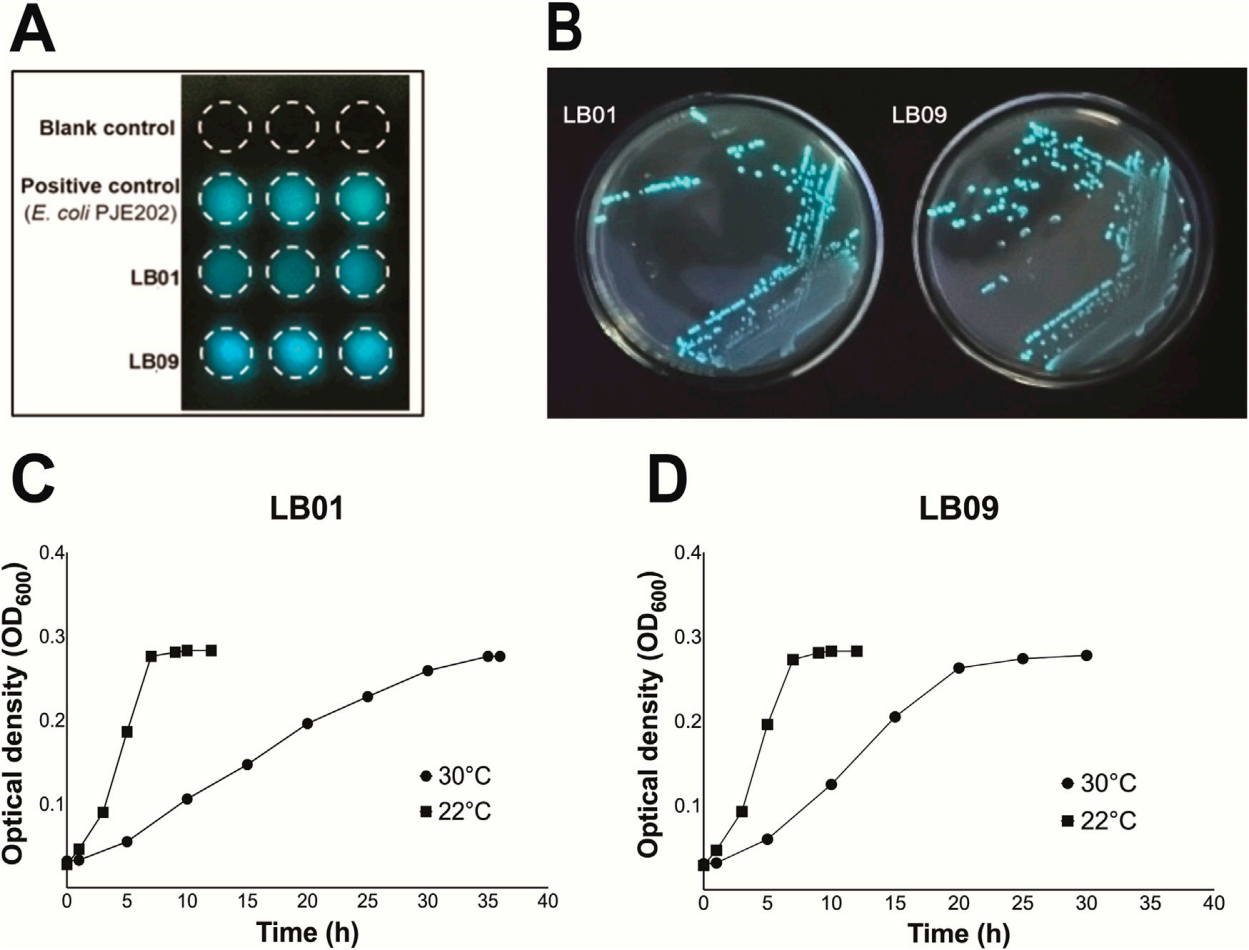
Bioluminescence assay and growth phases of *Phb. leiognathi* strains LB01 and LB09. **(A)** Bioluminescence assays. The mean specific bioluminescence (SB) was 6.04 E+06 for LB01, 1.04 E+07 for LB09, and 6.60 E+06 for the positive control (*E. coli* DH108 transformed with plasmid pJE202 expressing the Lux operon genes from *V. fisheri*. **(B)** LB01 and LB09 grown on seawater agar (SWA) plates after a 48-h incubation period at 28°C. **(C, D)** Growth phases of LB01 and LB09 at two distinct temperatures: 22°C and 30°C.

Two LB isolates, LB01 and LB09, identified as *Phb. leiognathi* subsp*. mandapemensis* and *Phb. leiognathi*, respectively, were selected based on their increased bioluminescence intensity after incubation at 28°C for 48 h (Figures 1A, B). The mean SB of LB01 was 6.04E+06, whereas LB09 exhibited a 1.7 times higher mean SB of 1.04E+7. This indicates that LB09 has a significantly higher bioluminescence capability than LB01. Nevertheless, both strains were studied further.

The growth dynamics of both strains were monitored at two distinct temperatures, 22°C and 30°C, to assess temperature-dependent growth variations (Figures 1C, D). At 22°C, both strains exhibited similar growth phases. The lag phase started <1 h and was immediately followed by the exponential phase. However, at 30°C, LB01 had a <2 h lag phase then followed by a prolonged exponential phase, which continued until approximately 32 h (Figure 1C). On the other hand, LB09 had a 1.5-h lag phase and a shorter exponential phase, which lasted until between approximately 22.5 h (Figure 1D). Both strains exhibited faster growth at the lower temperature.

### 3.2 *Phb. leiognathi* bioluminescence varied with GMPs concentration

**FIGURE 2:**
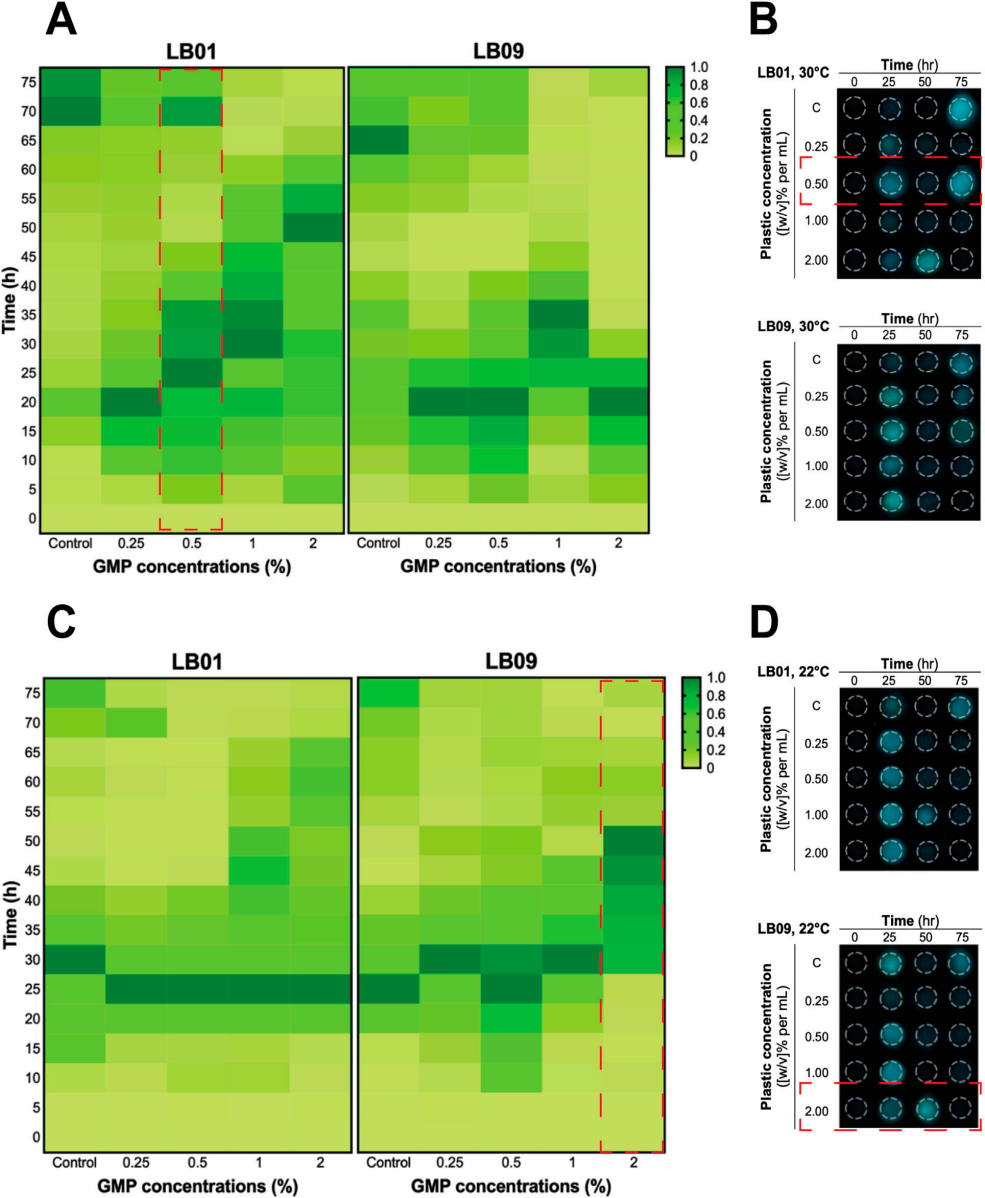
Heatmaps of the heterogenous bioluminescence responses of *Phb. leiognathi* strains LB01 and LB09 over 3.1 days at different ground microplastic (GMP) concentrations and two different temperatures, 30°C and 22°C. **(A)** At 30°C, prolonged bioluminescence activity of LB01 were observed with higher concentrations vs. lower GMP concentrations, indicating an enhanced response to GMPs, while the bioluminescence of LB09 exhibited shorter bioluminescence responses at this temperature, suggesting a possible inhibitory threshold. **(B)** Microtiter plate displaying bioluminescence of LB strains exposed to different GMP concentrations at 30°C and specified time points. Biphasic bioluminescence was observed in LB01 exposed to 0.5% GMP concentrations (in red broken lines). **(C)** At 22°C, both strains demonstrated uniform patterns of bioluminescence when compared to the control group. **(D)** Microtiter plate displaying bioluminescence of LB strains exposed to different GMP concentrations at 22°C and specified time points. LB09 exposed at a 2% GMP concentration exhibited prolonged and gradual bioluminescence response, as indicated by the red broken lines.

**FIGURE 3:**
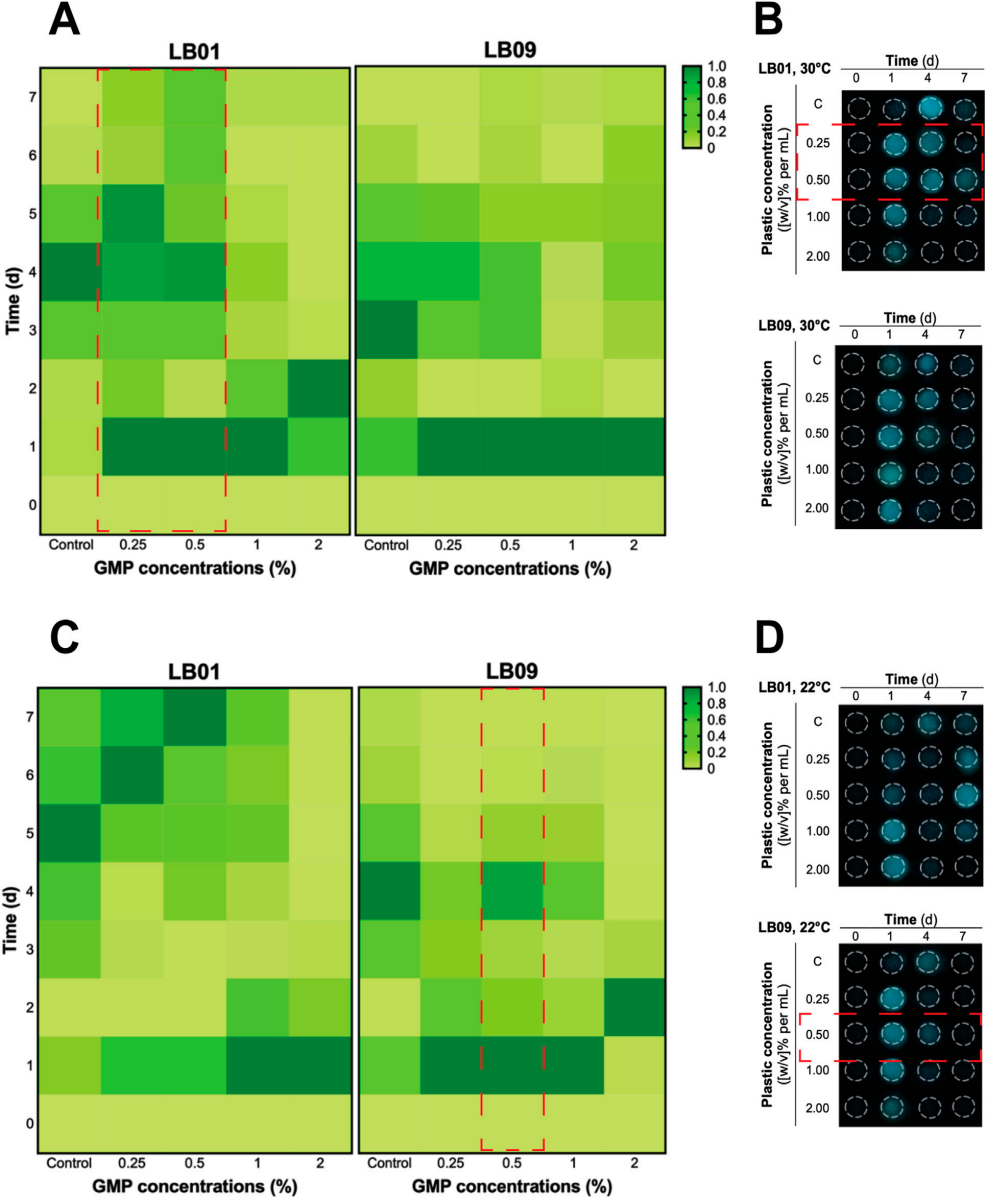
Heatmaps of the heterogenous bioluminescence responses of *Phb. leiognathi* strains LB01 and LB09 over 7 days at different ground microplastic (GMP) concentrations and two different temperatures, 30°C and 22°C. **(A)** At 30°C, both strains exhibited early pronounced bioluminescence compared to control groups. Biphasic bioluminescence patterns were observed in LB01 exposed to lower GMP concentrations (0.25% and 0.5%) (red broken lines). **(B)** Microtiter plate displaying bioluminescence of LB strains exposed to different GMP concentrations at 30°C and specified time points. **(C)** At 22°C, delayed bioluminescence was observed in LB01 exposed to lower GMP concentrations but earlier peaks at higher concentrations (1% and 2%). Potential biphasic bioluminescence pattern was observed in LB09 at 0.5% GMP concentration, as indicated in red broken lines. **(D)** Microtiter plate displaying bioluminescence of LB strains exposed to different GMP concentrations at 22°C and specified time points.

The effects of GMPs on the bioluminescence intensities of the two *Phb. leiognathi* strains at 22°C or 30°C were investigated. The SB values were presented in Supplementary Tables S1, S2). The bioluminescence intensities of the two strains exposed to varying GMP concentrations were found heterogeneous (Figures 2, 3).

In the 3.1-day experiment, which was used to assess the immediate effects, both strains displayed early peaked of bioluminescence intensity compared to the control group at 30°C (Figures 2A, B), suggesting an upregulation of QS in response to GMP exposure. Interestingly, a biphasic bioluminescence was observed in LB01 exposed at 0.5% GMP concentration that could reflect adjusted QS in response to the presence of GMPs (Figure 2A). These early bioluminescent responses of the two strains are indication of hormetic effect, where low-dose exposure triggers a stimulatory response. At 22°C, both strains exhibited uniform patterns of bioluminescence when compared to the control group, except for LB09 exposed to 2% GMP concentration (Figures 2C, D).

The 7-day experiment involved monitoring the bioluminescence of both strains at both temperatures, with varying GMP concentrations, which was used to assess the long-term effects of plastic particles (Figure 3). At 30°C, an obvious biphasic bioluminescence phenomenon was observed in LB01 at lower concentrations (0.25% and 0.5%) (Figure 3A). Furthermore, the strain also demonstrated biphasic-dose response, where biphasic bioluminescence observed at lower concentrations and a potential bioluminescence quenching (an inhibitory effect) at higher concentrations (1% and 2%) (Figure 3A). At 22°C, both strains showed complex bioluminescence responses (Figures 3C, D). LB01 exposed to lower concentrations showed delayed bioluminescence peaks (Figure 3C). In contrast, LB09 displayed initial bioluminescence peaks across all GMP concentrations, with a potential biphasic bioluminescence pattern observed specifically at the 0.5% GMP (Figure 3C).

Overall, these results highlight the heterogenous bioluminescence patterns of *Phb. leiognathi* strains to GMP exposure, with strain-specific responses emerging based in GMP concentration and temperature that lead to differences in their physiological adaptation. Furthermore, the observed biphasic bioluminescence and hormesis underscore the complex interactions between bacterial metabolic processes and MP stressors.

### 3.3 *Phb. leiognathi* demonstrated heterogenous recovery responses

**FIGURE 4:**
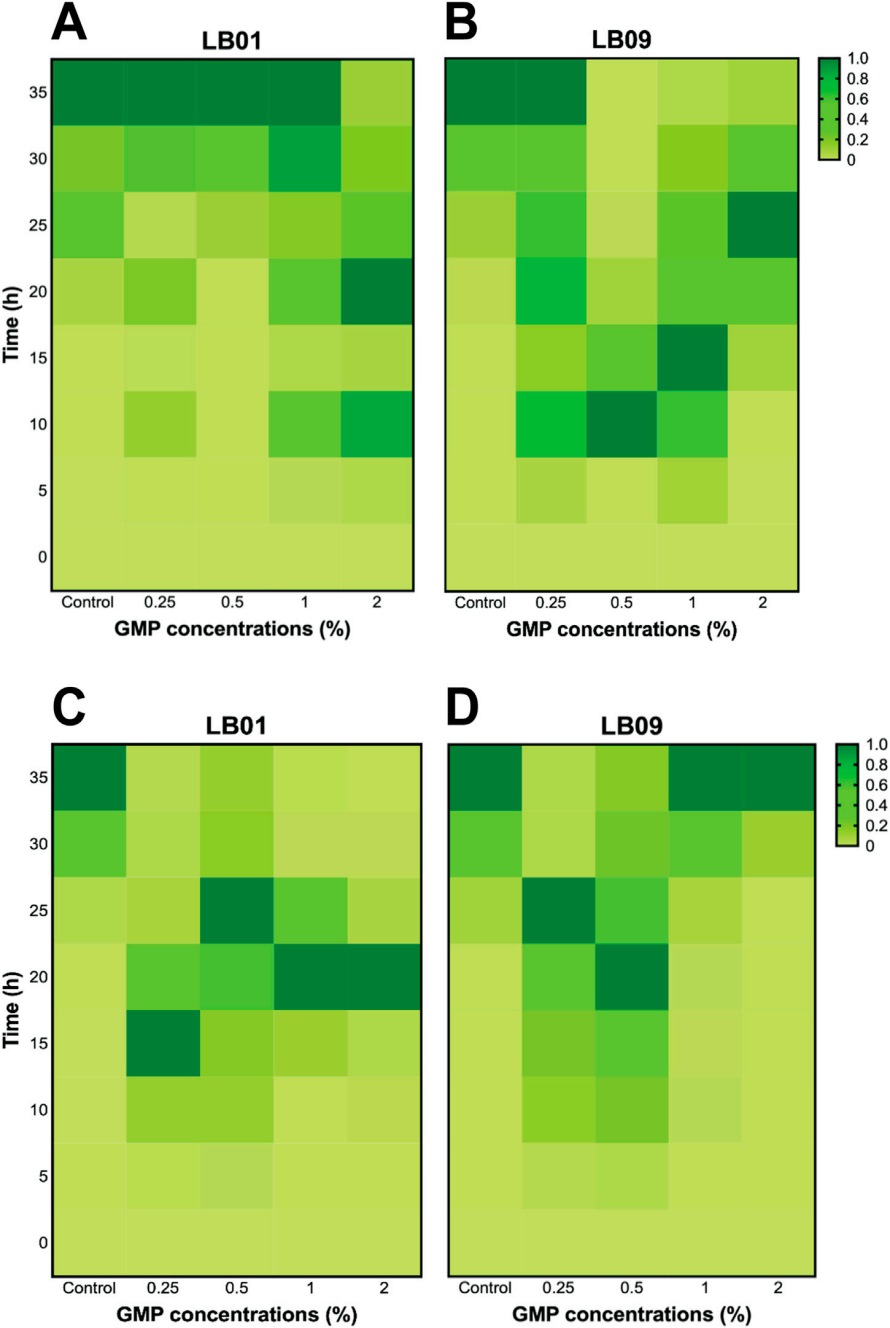
Heatmaps of the bioluminescence recovery patterns in *Photobacterium leiognathi* strains LB01 and LB09 following 3.1-day GMP exposure at 30°C **(A, B)** and 22°C **(C, D)**.

A post-exposure recovery experiment was conducted to determine whether the two strains previously exposed to GMPs for 3.1 days and 7 days could recuperate from the effects of this exposure. The SB values for the first 35 h were presented in Supplementary Tables S3, S4). Following the 3.1-day GMP exposure, the bioluminescence responses of LB01 exposed to all GMP concentrations were comparable to those of the control group, except at 2% GMP, indicating that the strain was able to recuperate from the effects of GMPs at low concentrations (Figure 4A). However, early bioluminescence peaks were observed in LB09 following exposure to 0.5%, 1%, and 2% GMPs, but the bacteria exposed to 0.25% displayed slower peaking of bioluminescence (Figure 4B). At 22°C, early bioluminescence peaks were still observed in LB01 following 3.1-day exposure to GMPs (Figure 4C), while LB09 showed contrasting responses, where the bacteria exposed at higher GMP concentrations have bioluminescence patterns comparable to the control group (Figure 4D).

**FIGURE 5:**
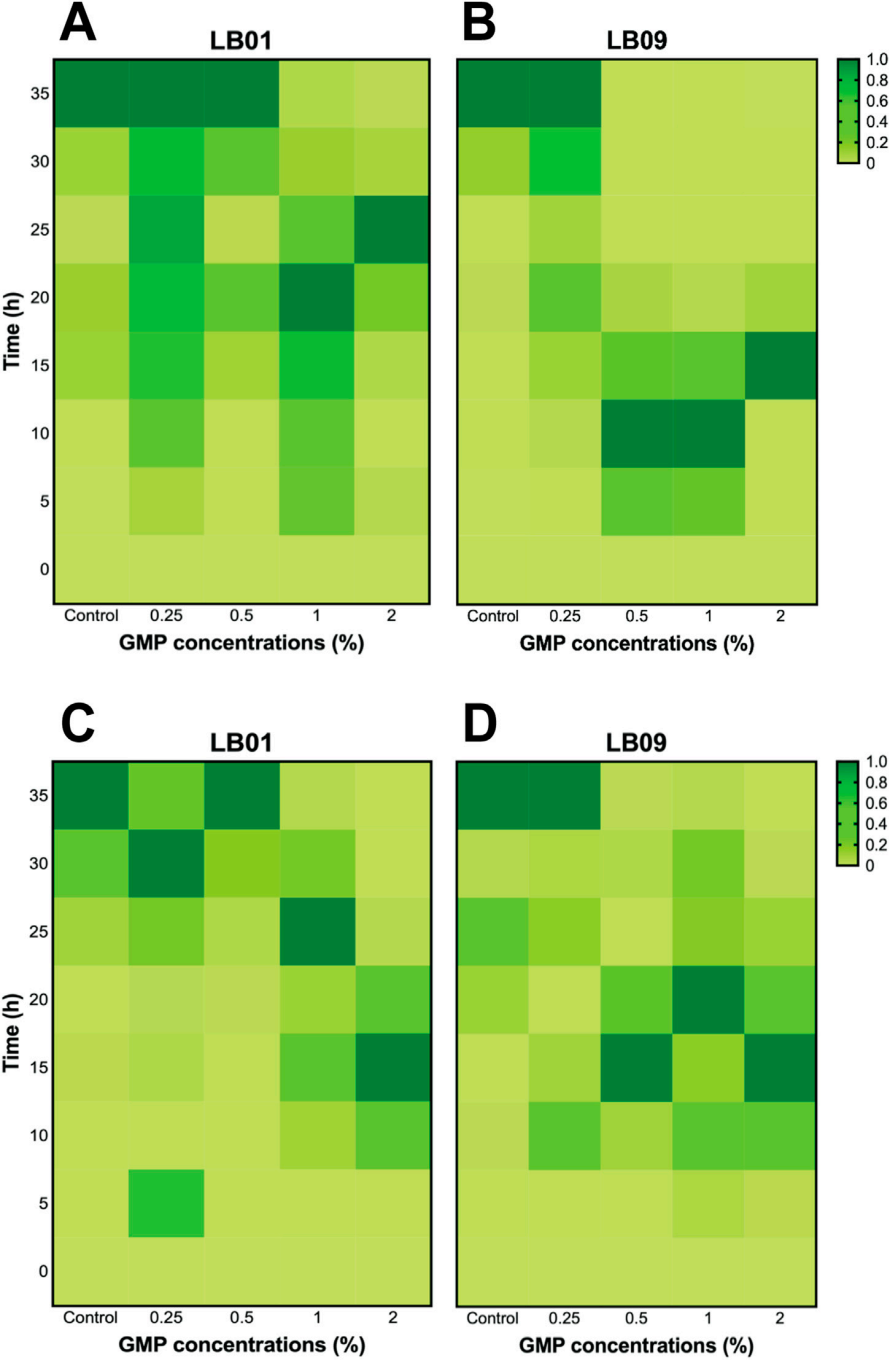
Heatmaps of the bioluminescence recovery patterns in *Photobacterium leiognathi* strains LB01 and LB09 following 7-day GMP exposure at 30°C **(A, B)** and 22°C **(C, D)**.

Following the 7 days GMP exposure, LB01 at 30°C displayed slower peaking of bioluminescence, while exposure to higher GMP concentrations (1% and 2%) remained incomparable with the control group (Figure 5A). Similarly, LB09 exposed to 0.25% GMP concentration at the same temperature also showed slower peaking of bioluminescence, but exposure to 0.5%, 1% and 2% GMP concentrations created initial peaks of bioluminescence (Figure 5B **)**. At 22°C, the bioluminescence responses of LB01 exposed to lower GMP concentrations were comparable to those of the control group (especially at 0.5% GMP concentration), except at 2%, indicating that the strain was able to recuperate from the effects of GMPs at low concentrations (Figure 5C). However, early bioluminescence peaks were observed in LB09 following exposure to 0.5%, 1%, and 2% GMPs, but the bacteria exposed to lowest GMP concentration also displayed slower peaking of bioluminescence (Figure 5D).

### 3.4 *Phb. leiognathi* viability varied with GMP concentrations

**FIGURE 6:**
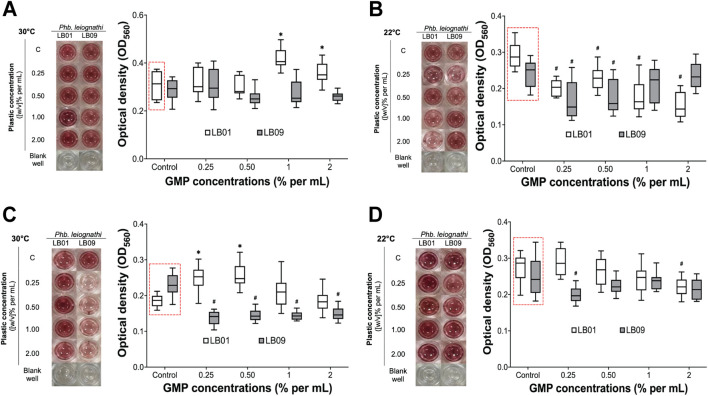
The cell viability of *Phb. leiognathi* strains LB01 and LB09 were assessed using MTT assays. Microtiter plate wells showing the production of red-violet formazan, a reduced form of MTT, after 8-h incubation. **(A, B)** LB following exposure to GMPs after 3.1 days at 30°C and 22°C, respectively. **(C, D)** LB following exposure to GMPs after 7 days at 30°C and 22°C, respectively. Control groups: **(C)**. Asterisks indicate significant increase in formazan production as compared to the control groups: *p < 0.05. Hashtags indicate significant decrease in formazan production as compared to the control groups: #*p* < 0.05. Error bars are the 95% confidence interval.

MTT assays were performed to evaluate the effects of GMPs on the cell viability of both strains under different experimental conditions (Figure 6). Then, the mean OD_560_ readings were determined and presented in Supplementary Tables S5, S6). In the 3.1-day experiment at 30°C, LB09 showed no significant decrease in viability compared to the control group (Figure 6A). LB01, where a hormetic effect of low GMP concentrations was previously observed, exhibited with no significant differences in viability at 0.25% and 0.50% GMP concentrations compared to the control groups (Figure 6A). At 22°C, LB01 revealed a significant (*p* < 0.05) decrease in viability after exposure at all GMP concentrations compared to the control group (Figure 6B). Conversely, LB09 had distinct MTT assays results at the same temperature. The viability decreased significantly (*p* < 0.05) at low concentrations (0.25% and 0.5%) compared to the control group, but not at high concentrations (1% and 2%), indicating possible adaptive mechanisms (Figure 6B).

Extending the exposure period to 7 days yielded substantial differences (Figures 6C, D). At 30°C, LB01 exhibited a significant (*p* < 0.05) increase in viability at low concentrations (0.25% and 0.5%) vs. to the control group, but not at high concentrations (1% and 2%) (Figure 6C). In contrast, LB09 demonstrated a significant (*p* < 0.05) decrease in viability compared to the control group at all concentrations (Figure 6C). At 22°C, LB01 showed a significant (*p* < 0.05) decrease in viability compared to the control groups at the 2% GMP concentration, whereas LB09 generally exhibited a decrease in viability at the 0.25% GMP concentration compared to control group (Figure 6D). These observations suggest strain-specific differences in bacterial viability to MPs through QS regulation, which influenced by GMP concentration, temperature, and time exposure.

### 3.5 Effects of GMPs in biofilm formation of *Phb. leiognathi* strains

**FIGURE 7:**
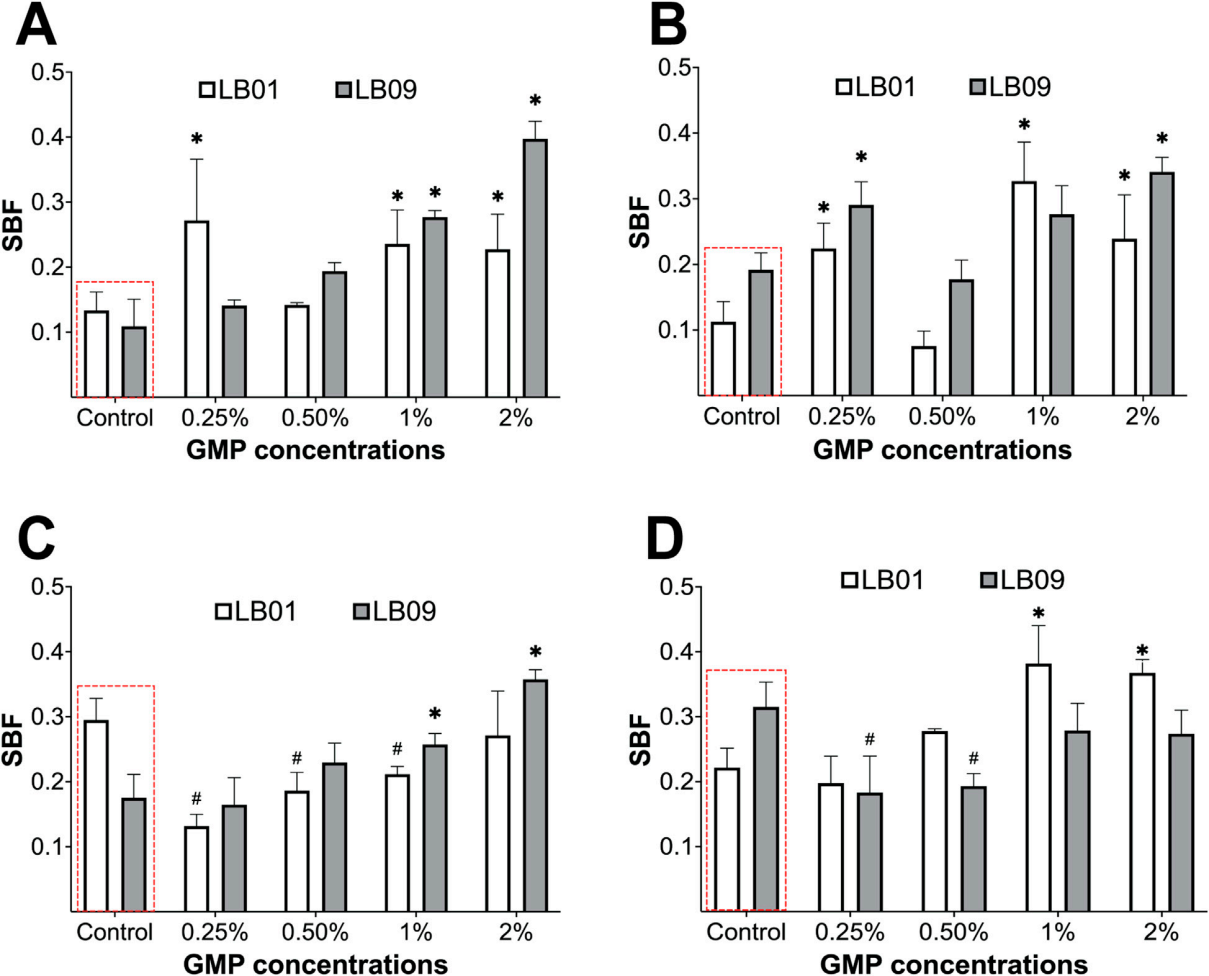
Biofilm formation of *Phb. leiognathi* strains LB01 and LB09 was assessed using crystal violet biofilm formation assays. **(A, B)** Biofilm formation of both strains exposed to GMPs vs. the control groups (red broken lines) for 3.1 days at 30°C and 22°C, respectively. **(C, D)** Biofilm formation of both strains exposed to GMPs vs. the control groups (red broken lines) for 7 days at 30°C and 22°C, respectively. Asterisks indicate significant increase in biofilm formation as compared to the control groups: *p < 0.05. Hashtags indicate significant decrease in biofilm formation as compared to the control groups: #*p* < 0.05. Error bars are the 95% confidence interval.

The biofilm formation of the two strains was assessed by crystal violet biofilm formation assays following GMP exposure (Figure 7). The biofilms were quantified and expressed as SBF, which were presented in Supplementary Tables S7, S8).

In the 3.1-day experiment at 30°C, the SBF of LB09 increased significantly (*p* < 0.05) as the GMP concentration increases compared to the control group (Figure 7A). Similarly, LB01 produced significantly (*p* < 0.05) high SBF at all GMP concentrations, except at 0.50%, compared to the control group (Figure 7A). On the other hand, at 22°C, the SBF in both strains increased vs. control group following exposure to GMP concentrations, except at 0.50% (Figure 7B). In the 7-day experiment, both strains showed contrasting SBF results at different temperatures (Figures 7C, D). It was observed that the LB01 and LB09 produced significantly (*p* < 0.05) low SBF as compared to the control group at 30°C and 22°C, respectively (Figures 7C, D). These observations suggest that there were strain-specific responses based on their biofilm formation after exposure to GMPs at lower temperature.

## 4 Discussion

The presence of plastics in the aquatic environment is widespread and varied. Studies have shown that 94% of the plastics entering the ocean settle on the sea floor, with an estimated 70 kg of plastics per square kilometer of seabed. About 1% of marine plastics are found floating at or near the ocean surface, with a global average concentration of less than 1 kg/km^2^. However, this concentration increases in certain mid-ocean locations, with the highest recorded concentration in the North Pacific Gyre at 18 kg/km^2^ (Author Anonymous, 2016). Plastic pollutants exist in ecosystems in various forms and sizes, which can be classified as megaplastics, macroplastics, mesoplastics, and MPs (Thushari and Senevirathna, 2020). Both primary MPs (produced directly as MPs) and secondary MPs (produced by the breakdown of larger plastic items) are widely distributed across marine and coastal environments, in both water, and sediments (Scherer et al., 2020; Lu et al., 2021). The concentrations of MPs in global marine and coastal ecosystems varies, ranging from 0.001 to 140 particles/m^3^ in water and from 0.2 to 8,766 particles/m^3^ in sediments globally (Thushari and Senevirathna, 2020; Quaglia et al., 2023; Veettil et al., 2024). These plastic particles can be ingested by fish and bivalves, causing physical harm, and potentially blocking digestive tracts, which can lead to starvation (Ryan, 2016; Egbeocha et al., 2018). Chemical pollutants from plastics can leach into the tissues of these animals, and the plastics can carry pathogens that increase the risk of disease (Gallo et al., 2018). In addition, marine LB, such as *Phb. leiognathi*, may exhibit impaired bioluminescence and cell viability due to plastic exposure, as observed in this study. Furthermore, biofilm formation on plastics can alter microbial communities within marine ecosystems (Pinto et al., 2019; Rummel et al., 2017; Zettler et al., 2013).

Bacterial bioluminescence is a complex mechanism regulated by QS that plays a vital role in both ecological and physiological processes (Anetzberger et al., 2009; Lupp et al., 2003). It has been reported that microbeads disrupted QS in bacterial populations by binding to autoinducer molecules (Gagné, 2017). The present study revealed intricate disparities in the bioluminescence patterns of *Phb. leiognathi* strains when exposed to different GMP concentrations at different temperatures. One of the critical observations in this study is the hormetic effect of low GMP concentrations in LB01 at 30°C in 3.1-day experiment. Hormesis is a dose–response phenomenon characterized by low-dose stimulation and high-dose inhibition (Calabrese and Baldwin, 2003), which is regulated by the QS system (Lin et al., 2023; Sun et al., 2018). Numerous studies have reported on the hormesis in various biota caused by environmental pollutants. Fan et al. (2021) reported on induced hormesis in soil microbial populations induced by cadmium and lead. Similar results were found for *Microcystis aeruginosa* exposed to halogenated organic pollutants (Zhang et al., 2022), *Phb. phosphoreum* exposed to sulfonamides (Deng et al., 2012), and plant species exposed to urban metal pollutants (Salinitro et al., 2021). Moreover, a recent meta-analysis on the effects of MPs at environmentally relevant concentrations (≤1 mg/L^-1^) on aquatic biota also revealed hormesis regarding various endpoints, such as behavior, genotoxicity, immunotoxicity, neurotoxicity, and reproduction (Sun et al., 2021).

Another critical observation in this study is the bioluminescence of high GMP concentrations in LB01 exposed for 7 days at 30°C. Several environmental contaminants exhibit inhibitory effects, resulting in various types of metabolic dysfunction. Aged tire wear particles, which are among the microplastic pollutants in the environment, were found to inhibit bacteria community leading to negatively affecting nitrogen metabolism in marine sediments (Liu et al., 2022). Aromatic compounds (such as benzene, toluene, and furfural) detected in wastewater were identified as growth inhibitors as they inhibited the metabolic assimilation processes of purple phototrophic bacteria (San Martín et al., 2021). It has been reported that membrane barrier impairment and direct inhibition of enzyme systems by toxic pollutants are likely to underlie bioluminescence quenching (Ismailov et al., 2000). In the present study, bioluminescence was not directly correlated with cell viability; while some conditions resulted in bioluminescence quenching, the *Phb. leiognathi* strains’ cells remained largely unaffected in terms of their ability to survive and grow (based on MTT assays). This discrepancy suggests that the factors influencing bioluminescence and cell viability are distinct and may involve different cellular mechanisms. Sully et al. (2014) found that the QS inhibitors disrupt bacterial behaviors, including biofilm formation, without directly killing or inhibiting *Staphylococcus aureus* cells. In addition, MPs are capable of adhering contaminants, which raises the possibility the MPs could also bind signaling molecules involved in QS resulting to disrupted QS mechanism (Gagné, 2017). Thus, bioluminescence alone may not be a reliable indicator of bacterial viability in this context.

Previous studies have reported the effects of plastic particles at different concentrations. For example, Liu et al. (2021) exposed *Streptomyces coelicolor* to nanoplastics and found that the fatality rate peaked (at 64.8%), when the particle size was 20 nm (tested range: 20 nm–1 mm) and the concentration was 10 mg/L (tested range: 0.1–10 mg/L). Tang et al. (2022) found that the polyethylene MPs at 100 and 500 MP/L shifted the community structure of sulfate-reducing bacteria. However, both *Phb. leiognathi* strains showed notably strain-specific response patterns, which highlights microbial variances in the presence of MPs. Yi et al. (2021) reported that 160 mg/L polystyrene microspheres, with sizes ranging from 0.323 to 0.656 µm, inhibited *E. coli* growth but promoted *Bacillus cereus* growth. They concluded that this difference is attributed to the cell wall compositions and surface interactions between each species and the polystyrene microspheres. The size of GMPs, ranging from 25.5 µm to 2.81 mm (Supplementary Figure S1). Larger particles (closer to 2.81 mm) may cause physical disruption by blocking signaling molecules or altering the microenvironment around the cells, which potentially hindering QS. In contrast, smaller particles (closer to 25.5 µm) have a larger surface area-to-volume ration, making them more likely to adsorb chemicals or release additives, which could affect bacterial metabolism more directly, resulting to bioluminescence alteration either through stress responses or metabolic interference. Both *Phb. leiognathi* strains used in the present study belong to the same genus and share similar cell wall compositions, so their differing “behaviors” under specific conditions suggest that other cellular functions may influence their responses to GMPs.

The post-exposure recovery experiments conducted in this study demonstrated that both strains exhibited heterogenous recovery responses possibly that the response dynamics change within 3-day and 7-day GMPs exposure due to the alteration in gene expression, which eventually affecting QS mechanism responsible for bioluminescence. For instance, genes regulating light production can be upregulated or downregulated in response to environmental stress, shifting in the timing of peak bioluminescence (Tu et al., 2008; de Nadal et al., 2011; Zavilgelsky et al., 2015). Additionally, this study suggests that the bioluminescence response to GMP exposure in *Phb. leiognathi* strains is concentration-, time-, and temperature-dependent, which emphasize the importance of temporal and physical factors on evaluating the environmental impacts of pollutants on marine organisms. The bacterial response from GMPs might influences QS mechanisms, which are essential for coordinating bioluminescence in bacterial populations. Plastic particles may influence signaling molecules that bacteria use to communicate, resulting in a collective bioluminescence response (Gagné, 2017). The premature bioluminescence activities observed in this study emphasize the need for further studies to understand the full ecophysiological consequences of plastic exposure. Future research should focus on elucidating the genetic and molecular mechanisms underlying these responses.

The present study indicates that biofilm formation in *Phb. leiognathi* strains LB01 and LB09 is influenced by exposure to different GMP concentrations, temperature, and exposure duration. The crystal violet biofilm formation assays revealed distinct biofilm development under varying conditions, suggesting complex interactions between the set laboratory conditions and bacterial behavior. The increased biofilm formation observed with higher concentrations vs. lower GMP concentrations was due to bacteria utilizing the GMPs as a substrate, which promoted adherence and aggregation of bacterial cells. Plastics have been reported to be utilized by bacteria as substrates and they induce bacterial aggregation (Ganesan et al., 2022; Hossain et al., 2018; Ayush et al., 2022). For instance, plastics with reduced hydrophobicity increased *E. coli* biofilm formation and exopolysaccharide content (Ganesan et al., 2022). Conversely, both strains have opposite biofilm formation responses in 7-day experiment at 22°C. These observations may be attributable to the differential metabolic responses of the bacteria at low temperature (Price and Sowers, 2004). Additionally, the zeta potential of plastic particles plays a significant role in biofilm formation (Ganesan et al., 2022; Okshevsky et al., 2020; Saygin and Baysal, 2020). A more negative zeta potential generally enhances bacterial adhesion by reducing electrostatic repulsion between bacterial cells and the plastic surface (Abram et al., 2021). This effect was particularly evident at higher GMP concentrations in the present study. Furthermore, the increased biofilm production observed in this study corroborated previous findings that plastics provide additional surface area that facilitates biofilm growth (Ganesan et al., 2022; Webb et al., 2009; Semcesen and Wells, 2021). However, at 22°C in 7-day experiment, the results for LB01 and LB09 differed, pointing again to strain-specific responses to GMPs. The increase in biofilm formation with higher GMPs, especially at 30°C and over longer exposure periods, suggests that temperature and exposure duration are critical factors in biofilm dynamics. Higher temperatures generally enhance microbial activity and biofilm stability, while longer exposure times allow bacteria to adapt and optimize their biofilm-forming capabilities (Stepanović et al., 2003; Bhagwat et al., 2021; Tu et al., 2021). These findings align with studies that have shown that biofilm formation is a complex adaptive response to environmental conditions, which includes factors such as nutrient availability, temperature, and the presence of surfaces for attachment.

The environmental implications of our findings are significant, given the escalating concerns surrounding plastic pollution in marine ecosystems. The present study suggests that plastic particles, prevalent in marine environments, may alter the natural bioluminescence behavior of *Phb. leiognathi* potentially impacting ecological interactions and energy transfer in these ecosystems, which can be applied to explore the combined effects of multiple pollutants, on microbial physiology in real marine environments. For instance, xenobiotics may alter bacterial biofilm formation, quorum sensing, and bioluminescence (Gagné, 2017; Kumari et al., 2016), as well as microbial interactions with MPs. On the other hand, this research will benefit environmental scientists, marine biologists, and policymakers by offering insights into the ecophysiological repercussions of MP pollution. Understanding these interactions opens potential avenues for biotechnological applications, such as developing sensitive bioindicators for monitoring environmental pollution levels, including the MPs. For future research, exploring longer exposure durations, investigating gene regulation changes underlying the cellular mechanisms, using different types of plastics, and simulating more natural conditions to reflect the full complexity of marine environments and MP interactions are warranted to better understand the ecophysiological consequences of MP pollution.

## Data Availability

The datasets presented in this study can be found in online repositories. The names of the repository/repositories and accession number(s) can be found in the article/Supplementary Material.
