# Supplementary material for: Heterogenous bioluminescence patterns, cell viability, and biofilm formation of Photobacterium leiognathi strains exposed to ground microplastics
Source: Front Toxicol. 2024 Nov 27;6:1479549. doi: 10.3389/ftox.2024.1479549 (PMC11631867; doi:10.3389/ftox.2024.1479549)
Supplement: Supplementary file 1 [file Table1.docx]

Supplementary Material

# Supplementary Tables

The bioluminescence data, which expressed as specific bioluminescence (SBF), includes measurements recorded in Log relative light units (Log RLUs) using Glomax Discover (Promega Instrument). These measurements were taken at multiple time intervals, such as every 5 h for 3.1 days (75 h) or 24 h for 7 days, to capture the temporal changes in bioluminescence activity during and after (post-exposure experiment) exposure to GMPs (Tables S1-S4). For cell viability, the data represented as optical density (ODs) at 560 nm. MTT assays were conducted to determine cell viability. The wells were added with MTT solution (5 mg/mL concentration), incubated for 8 h and dissolved with dimethyl sulfoxide. This analysis enabling an examination of the impact of GMPs on bacterial growth and survival (Tables S5-S6). Biofilm formation data was obtained by quantifying the biofilm biomass using 1% crystal violet solution. This involved measuring the ODs at 570 nm to determine the amount of biofilm produced by *Phb. leiognathi* under each condition. The dataset includes biofilm measurements for both the control (unexposed to GMPs) and experimental groups, corresponding to the same GMP concentrations used in the bioluminescence and cell viability experiments (Tables S7-S8). This dataset is valuable for researchers studying the environmental effects of microplastics on marine bacteria, particularly those interested in the functional responses of bioluminescent organisms to plastic pollution. The comprehensive data on bioluminescence, cell viability, and biofilm formation provides a robust foundation for understanding the ecophysiological effects of GMPs to *Phb. leiognathi*.

## *Phb. leiognathi* Bioluminescence varied with GMP concentrations

The effects of GMPs on the bioluminescence intensities of the *Phb. leiognathi* strains LB01 and LB02 at 22°C or 30°C were investigated. The SB values are presented in Tables S1 and S2.

**Table S1.** The mean SB values (Log RLU) of *Phb. leiognathi* strains for 3.1 days GMP exposure.

| Temperature: 30°C | | | | | | | | | | |
| --- | --- | --- | --- | --- | --- | --- | --- | --- | --- | --- |
| Time (h) | LB_01 | | | | | LB_09 | | | | |
|  | Control | GMP concentrations (w/v per mL) | | | | Control | GMP concentrations (w/v per mL) | | | |
|  |  | 0.25% | 0.5% | 1% | 2% |  | 0.25% | 0.5% | 1% | 2% |
| 0 | 1.43E+02 | 1.41E+02 | 1.43E+02 | 1.44E+02 | 1.44E+02 | 1.39E+02 | 1.35E+02 | 1.42E+02 | 1.36E+02 | 1.38E+02 |
| 5 | 5.22E+03 | 5.47E+04 | 9.11E+04 | 5.16E+04 | 1.53E+05 | 1.28E+04 | 1.74E+05 | 1.90E+05 | 1.40E+05 | 2.76E+05 |
| 10 | 5.36E+03 | 4.11E+05 | 3.00E+05 | 2.66E+05 | 7.29E+04 | 5.75E+04 | 6.16E+05 | 5.43E+05 | 6.03E+04 | 6.41E+05 |
| 15 | 4.93E+04 | 7.20E+05 | 3.26E+05 | 3.92E+05 | 1.66E+05 | 1.28E+05 | 1.36E+06 | 6.78E+05 | 2.99E+05 | 1.14E+06 |
| 20 | 9.31E+04 | 1.03E+06 | 3.53E+05 | 5.20E+05 | 2.63E+05 | 2.00E+05 | 2.08E+06 | 8.19E+05 | 5.35E+05 | 1.64E+06 |
| 25 | 3.41E+04 | 5.17E+05 | 4.83E+05 | 3.15E+05 | 2.64E+05 | 1.55E+05 | 1.37E+06 | 5.66E+05 | 1.37E+06 | 1.31E+06 |
| 30 | 2.05E+04 | 2.41E+05 | 4.25E+05 | 6.67E+05 | 2.76E+05 | 1.17E+05 | 3.90E+05 | 2.72E+05 | 1.56E+06 | 2.41E+05 |
| 35 | 1.48E+04 | 1.89E+05 | 4.32E+05 | 6.49E+05 | 2.15E+05 | 1.41E+05 | 1.66E+05 | 2.99E+05 | 1.71E+06 | 3.81E+04 |
| 40 | 1.67E+04 | 1.41E+05 | 2.62E+05 | 5.53E+05 | 1.95E+05 | 7.89E+04 | 8.87E+04 | 1.52E+05 | 9.88E+05 | 2.06E+04 |
| 45 | 1.75E+04 | 9.46E+04 | 9.22E+04 | 4.69E+05 | 1.74E+05 | 1.61E+04 | 1.19E+04 | 6.49E+03 | 2.64E+05 | 5.48E+03 |
| 50 | 2.66E+04 | 1.19E+05 | 1.78E+04 | 3.64E+05 | 4.26E+05 | 3.39E+04 | 9.48E+03 | 5.41E+03 | 8.41E+04 | 5.87E+03 |
| 55 | 3.89E+04 | 1.33E+05 | 2.21E+04 | 2.79E+05 | 3.55E+05 | 9.19E+04 | 2.76E+05 | 3.55E+04 | 5.54E+04 | 8.20E+03 |
| 60 | 5.45E+04 | 1.60E+05 | 5.48E+04 | 9.38E+04 | 1.17E+05 | 2.75E+05 | 4.05E+05 | 8.08E+04 | 3.64E+04 | 1.29E+04 |
| 65 | 5.63E+04 | 1.60E+05 | 5.98E+04 | 1.02E+04 | 4.80E+04 | 5.20E+05 | 5.31E+05 | 2.05E+05 | 2.96E+04 | 1.92E+04 |
| 70 | 3.27E+05 | 3.97E+05 | 4.30E+05 | 1.76E+04 | 1.39E+04 | 3.06E+05 | 3.85E+05 | 3.55E+05 | 4.57E+04 | 2.06E+04 |
| 75 | 3.01E+05 | 2.48E+05 | 2.35E+05 | 5.09E+04 | 7.41E+03 | 2.51E+05 | 7.30E+05 | 3.42E+05 | 3.99E+04 | 1.42E+05 |

**Table S1.** (Continuation)

| Temperature: 22°C | | | | | | | | | | |
| --- | --- | --- | --- | --- | --- | --- | --- | --- | --- | --- |
| Time (h) | LB_01 | | | | | LB_09 | | | | |
|  | Control | GMP concentrations (w/v per mL) | | | | Control | GMP concentrations (w/v per mL) | | | |
|  |  | 0.25% | 0.5% | 1% | 2% |  | 0.25% | 0.5% | 1% | 2% |
| 0 | 1.42E+02 | 1.41E+02 | 1.41E+02 | 1.40E+02 | 1.41E+02 | 1.35E+02 | 1.41E+02 | 1.41E+02 | 1.42E+02 | 1.39E+02 |
| 5 | 2.16E+02 | 1.77E+02 | 6.07E+02 | 2.23E+02 | 2.76E+02 | 1.41E+02 | 2.92E+02 | 1.62E+02 | 2.11E+02 | 3.18E+02 |
| 10 | 7.36E+03 | 9.46E+03 | 2.64E+04 | 5.36E+04 | 3.35E+03 | 1.71E+03 | 6.40E+03 | 1.52E+04 | 8.13E+03 | 1.41E+04 |
| 15 | 5.56E+04 | 2.27E+04 | 1.83E+04 | 4.10E+04 | 5.55E+03 | 2.37E+03 | 2.18E+04 | 3.14E+04 | 1.57E+04 | 8.16E+03 |
| 20 | 5.80E+04 | 1.27E+05 | 1.02E+05 | 2.10E+05 | 5.59E+04 | 4.62E+04 | 4.83E+04 | 3.93E+04 | 8.00E+04 | 1.16E+04 |
| 25 | 6.29E+04 | 3.37E+05 | 2.69E+05 | 5.47E+05 | 1.57E+05 | 1.34E+05 | 1.01E+05 | 5.52E+04 | 2.09E+05 | 1.84E+04 |
| 30 | 1.49E+05 | 1.36E+05 | 9.42E+04 | 2.22E+05 | 4.79E+04 | 7.25E+04 | 1.94E+05 | 5.14E+04 | 5.49E+05 | 4.99E+05 |
| 35 | 7.23E+04 | 7.32E+04 | 6.83E+04 | 2.71E+05 | 4.11E+04 | 3.32E+04 | 9.45E+04 | 2.83E+04 | 3.56E+05 | 5.15E+05 |
| 40 | 3.39E+04 | 4.16E+04 | 5.54E+04 | 3.17E+05 | 3.77E+04 | 1.36E+04 | 4.50E+04 | 1.67E+04 | 2.59E+05 | 5.41E+05 |
| 45 | 6.94E+03 | 1.96E+03 | 5.24E+03 | 3.69E+05 | 3.57E+04 | 1.83E+03 | 1.53E+04 | 9.77E+03 | 2.02E+05 | 5.94E+05 |
| 50 | 2.81E+03 | 1.26E+03 | 2.47E+03 | 3.19E+05 | 3.11E+04 | 1.87E+03 | 3.21E+04 | 1.06E+04 | 2.14E+04 | 6.36E+05 |
| 55 | 2.61E+03 | 4.51E+03 | 2.72E+03 | 7.02E+04 | 7.19E+04 | 2.61E+03 | 4.51E+03 | 2.72E+03 | 7.02E+04 | 7.19E+04 |
| 60 | 1.13E+04 | 6.05E+03 | 3.46E+03 | 8.85E+04 | 9.29E+04 | 1.13E+04 | 6.05E+03 | 3.46E+03 | 8.85E+04 | 9.29E+04 |
| 65 | 5.50E+03 | 5.02E+03 | 2.97E+03 | 6.41E+04 | 6.49E+04 | 2.00E+04 | 7.60E+03 | 5.67E+03 | 5.18E+04 | 5.10E+04 |
| 70 | 2.87E+04 | 9.14E+03 | 4.93E+03 | 1.51E+04 | 9.01E+03 | 2.87E+04 | 9.14E+03 | 4.93E+03 | 1.51E+04 | 9.01E+03 |
| 75 | 8.86E+04 | 1.71E+04 | 4.97E+03 | 1.25E+04 | 6.42E+03 | 8.86E+04 | 1.71E+04 | 4.97E+03 | 6.91E+03 | 4.04E+04 |

**Table S2.** The mean SB values (Log RLU) of *Phb. leiognathi* strains for 7 days GMP exposure.

| Temperature: 30°C | | | | | | | | | | |
| --- | --- | --- | --- | --- | --- | --- | --- | --- | --- | --- |
| Time (d) | LB_01 | | | | | LB_09 | | | | |
|  | Control | GMP concentrations (w/v per mL) | | | | Control | GMP concentrations (w/v per mL) | | | |
|  |  | 0.25% | 0.5% | 1% | 2% |  | 0.25% | 0.5% | 1% | 2% |
| 0 | 1.43E+02 | 1.41E+02 | 1.43E+02 | 1.44E+02 | 1.42E+02 | 1.39E+02 | 1.35E+02 | 1.42E+02 | 1.36E+02 | 1.38E+02 |
| 1 | 3.41E+04 | 5.66E+05 | 5.14E+05 | 6.67E+05 | 2.64E+05 | 1.55E+05 | 1.37E+06 | 5.66E+05 | 1.37E+06 | 1.31E+06 |
| 2 | 2.66E+04 | 1.19E+05 | 1.78E+04 | 3.64E+05 | 4.26E+05 | 3.39E+04 | 9.48E+03 | 5.41E+03 | 8.41E+04 | 5.87E+03 |
| 3 | 3.01E+05 | 2.48E+05 | 2.35E+05 | 5.09E+04 | 7.41E+03 | 2.51E+05 | 7.30E+05 | 3.42E+05 | 3.99E+04 | 1.42E+05 |
| 4 | 6.01E+05 | 4.96E+05 | 4.71E+05 | 1.02E+05 | 5.62E+03 | 2.01E+05 | 1.07E+06 | 3.29E+05 | 3.43E+04 | 2.62E+05 |
| 5 | 1.89E+05 | 5.32E+05 | 1.21E+05 | 4.02E+04 | 5.80E+03 | 1.23E+05 | 3.43E+05 | 7.96E+04 | 2.18E+05 | 2.36E+05 |
| 6 | 2.16E+04 | 6.48E+04 | 1.79E+05 | 6.13E+03 | 3.37E+03 | 2.17E+04 | 5.65E+03 | 4.39E+04 | 1.43E+04 | 1.84E+05 |
| 7 | 7.15E+03 | 8.62E+04 | 1.97E+05 | 2.78E+04 | 1.85E+04 | 1.36E+03 | 3.57E+03 | 3.40E+04 | 4.73E+04 | 6.93E+04 |
|  | Temperature: 22°C | | | | | | | | | |
| 0 | 1.42E+02 | 1.41E+02 | 1.41E+02 | 1.40E+02 | 1.41E+02 | 1.35E+02 | 1.41E+02 | 1.41E+02 | 1.42E+02 | 1.39E+02 |
| 1 | 6.29E+04 | 3.37E+05 | 2.69E+05 | 5.47E+05 | 1.57E+05 | 1.34E+05 | 1.01E+05 | 5.52E+04 | 2.09E+05 | 1.84E+04 |
| 2 | 2.81E+03 | 1.26E+03 | 2.47E+03 | 3.19E+05 | 3.11E+04 | 1.87E+03 | 3.21E+04 | 1.06E+04 | 2.14E+04 | 6.36E+05 |
| 3 | 8.86E+04 | 1.71E+04 | 4.97E+03 | 1.25E+04 | 6.42E+03 | 8.86E+04 | 1.71E+04 | 4.97E+03 | 6.91E+03 | 4.04E+04 |
| 4 | 2.16E+05 | 9.06E+03 | 8.74E+04 | 3.89E+04 | 9.36E+02 | 2.65E+05 | 2.11E+04 | 4.78E+04 | 7.33E+04 | 1.87E+03 |
| 5 | 3.67E+05 | 1.38E+05 | 1.10E+05 | 1.36E+05 | 1.05E+02 | 1.40E+05 | 4.05E+03 | 7.26E+03 | 2.44E+04 | 1.19E+02 |
| 6 | 2.26E+05 | 5.24E+05 | 1.22E+05 | 1.09E+05 | 8.17E+02 | 2.37E+04 | 4.48E+02 | 1.01E+03 | 5.31E+03 | 1.74E+03 |
| 7 | 1.43E+05 | 4.36E+05 | 4.11E+05 | 1.45E+05 | 1.99E+03 | 1.12E+04 | 2.41E+02 | 5.27E+02 | 1.94E+03 | 3.37E+02 |

## *Phb. leiognathi* demonstrated heterogenous recovery responses

A post-exposure recovery experiment was conducted to determine whether the two strains previously exposed to GMPs for 3.1 days and 7 days could recuperate from the effects of this exposure. The SB values are presented in Tables S3 and S4.

**Table S3.** The mean SB values (Log RLU) of *Phb. leiognathi* strains after 3.1 days GMP exposure.

| Temperature: 30°C | | | | | | | | | | |
| --- | --- | --- | --- | --- | --- | --- | --- | --- | --- | --- |
| Time (h) | LB_01 | | | | | LB_09 | | | | |
|  | Control | GMP concentrations (w/v per mL) | | | | Control | GMP concentrations (w/v per mL) | | | |
|  |  | 0.25% | 0.5% | 1% | 2% |  | 0.25% | 0.5% | 1% | 2% |
| 0 | 7.87E+01 | 7.83E+01 | 7.73E+01 | 7.73E+01 | 7.77E+01 | 7.90E+01 | 7.57E+01 | 7.70E+01 | 7.87E+01 | 7.73E+01 |
| 5 | 2.11E+02 | 1.24E+02 | 2.57E+02 | 1.32E+02 | 3.53E+02 | 6.11E+02 | 8.03E+02 | 1.68E+02 | 3.51E+02 | 8.03E+01 |
| 10 | 6.92E+02 | 1.91E+03 | 4.58E+02 | 7.99E+02 | 5.66E+03 | 1.73E+03 | 7.83E+03 | 1.19E+04 | 1.82E+03 | 1.51E+02 |
| 15 | 1.51E+03 | 2.97E+02 | 6.61E+02 | 1.88E+02 | 5.16E+02 | 3.36E+03 | 1.80E+03 | 4.57E+03 | 2.85E+03 | 3.40E+03 |
| 20 | 2.41E+04 | 2.98E+03 | 1.24E+03 | 9.43E+02 | 6.70E+03 | 1.14E+04 | 8.71E+03 | 1.10E+03 | 1.32E+03 | 1.52E+04 |
| 25 | 1.37E+05 | 5.67E+02 | 1.52E+04 | 4.69E+02 | 1.86E+03 | 4.27E+04 | 7.05E+03 | 3.03E+02 | 8.20E+02 | 3.63E+04 |
| 30 | 8.31E+04 | 8.46E+03 | 4.63E+04 | 2.10E+03 | 1.33E+03 | 1.34E+05 | 4.40E+03 | 1.76E+02 | 5.49E+02 | 9.98E+03 |
| 35 | 3.64E+05 | 1.48E+04 | 1.34E+05 | 2.39E+03 | 7.83E+02 | 3.86E+05 | 1.12E+04 | 1.41E+02 | 2.02E+02 | 3.04E+03 |
| Temperature: 22°C | | | | | | | | | | |
| 0 | 7.83E+01 | 7.77E+01 | 7.80E+01 | 7.87E+01 | 7.73E+01 | 7.80E+01 | 7.67E+01 | 7.77E+01 | 7.67E+01 | 7.63E+01 |
| 5 | 2.74E+03 | 1.31E+02 | 1.47E+02 | 1.46E+02 | 2.08E+02 | 5.65E+02 | 1.48E+02 | 1.68E+02 | 2.52E+02 | 3.23E+02 |
| 10 | 2.49E+02 | 4.93E+02 | 4.26E+02 | 1.71E+02 | 4.20E+02 | 9.73E+02 | 3.92E+02 | 5.47E+02 | 3.87E+03 | 6.54E+02 |
| 15 | 3.26E+02 | 3.23E+03 | 5.49E+02 | 8.97E+02 | 6.72E+02 | 1.99E+03 | 5.85E+02 | 9.36E+02 | 2.96E+03 | 1.55E+03 |
| 20 | 4.01E+03 | 1.37E+03 | 1.72E+03 | 7.04E+03 | 1.24E+04 | 4.16E+03 | 8.46E+02 | 2.15E+03 | 4.04E+03 | 2.02E+03 |
| 25 | 2.63E+04 | 3.10E+02 | 2.81E+03 | 2.34E+03 | 8.55E+02 | 4.57E+04 | 2.30E+03 | 1.30E+03 | 1.24E+04 | 3.89E+03 |
| 30 | 2.54E+05 | 2.20E+02 | 5.09E+02 | 2.03E+02 | 3.20E+02 | 2.78E+05 | 1.85E+02 | 5.67E+02 | 5.27E+04 | 4.54E+04 |
| 35 | 6.14E+05 | 1.80E+02 | 4.08E+02 | 1.96E+02 | 1.96E+02 | 5.30E+05 | 1.75E+02 | 4.37E+02 | 1.59E+05 | 3.84E+05 |

**Table S4.** The mean SB values (Log RLU) of *Phb. leiognathi* strains after 7 days GMP exposure.

| Temperature: 30°C | | | | | | | | | | |
| --- | --- | --- | --- | --- | --- | --- | --- | --- | --- | --- |
| Time (h) | LB_01 | | | | | LB_09 | | | | |
|  | Control | GMP concentrations (w/v per mL) | | | | Control | GMP concentrations (w/v per mL) | | | |
|  |  | 0.25% | 0.5% | 1% | 2% |  | 0.25% | 0.5% | 1% | 2% |
| 0 | 7.80E+01 | 7.70E+01 | 7.67E+01 | 7.87E+01 | 7.87E+01 | 7.70E+01 | 7.83E+01 | 7.83E+01 | 7.80E+01 | 7.73E+01 |
| 5 | 4.37E+02 | 6.34E+02 | 1.17E+03 | 6.86E+02 | 4.86E+02 | 2.97E+02 | 1.62E+03 | 2.48E+03 | 5.17E+03 | 4.94E+03 |
| 10 | 2.16E+04 | 2.13E+03 | 3.23E+03 | 8.44E+02 | 1.38E+02 | 1.18E+04 | 1.38E+04 | 7.08E+04 | 2.06E+04 | 3.20E+04 |
| 15 | 3.77E+05 | 4.52E+03 | 2.32E+04 | 1.67E+03 | 5.32E+02 | 5.31E+03 | 3.80E+04 | 1.87E+04 | 8.72E+03 | 4.73E+06 |
| 20 | 4.50E+05 | 5.33E+03 | 6.68E+04 | 2.42E+03 | 2.38E+03 | 5.70E+04 | 1.14E+05 | 4.65E+03 | 8.00E+02 | 3.91E+05 |
| 25 | 8.58E+04 | 6.07E+03 | 6.53E+03 | 1.02E+03 | 1.09E+04 | 1.69E+04 | 3.25E+04 | 7.15E+02 | 2.20E+02 | 6.26E+04 |
| 30 | 3.77E+05 | 4.98E+03 | 8.76E+04 | 3.54E+02 | 8.28E+02 | 3.25E+05 | 2.52E+05 | 7.85E+02 | 3.30E+02 | 3.72E+03 |
| 35 | 3.77E+06 | 7.04E+03 | 2.35E+05 | 1.74E+02 | 2.89E+02 | 2.46E+06 | 3.80E+05 | 2.55E+02 | 1.44E+02 | 7.76E+02 |
| Temperature: 22°C | | | | | | | | | | |
| 0 | 7.77E+01 | 7.80E+01 | 7.67E+01 | 7.73E+01 | 7.83E+01 | 7.80E+01 | 7.63E+01 | 7.80E+01 | 7.70E+01 | 7.87E+01 |
| 5 | 2.08E+03 | 1.50E+02 | 1.83E+02 | 1.47E+02 | 2.69E+02 | 1.75E+03 | 1.46E+03 | 1.96E+03 | 8.72E+03 | 3.88E+02 |
| 10 | 1.98E+04 | 1.16E+03 | 5.89E+02 | 1.25E+03 | 4.25E+03 | 1.06E+04 | 3.57E+04 | 1.84E+04 | 6.58E+04 | 3.77E+03 |
| 15 | 6.95E+04 | 1.58E+04 | 9.92E+02 | 5.84E+03 | 1.53E+04 | 2.15E+03 | 1.13E+04 | 1.98E+05 | 2.21E+04 | 1.09E+04 |
| 20 | 2.50E+04 | 9.50E+03 | 2.81E+03 | 1.37E+03 | 7.42E+03 | 5.47E+04 | 1.57E+03 | 5.24E+04 | 1.56E+05 | 3.77E+03 |
| 25 | 2.24E+05 | 7.68E+04 | 7.50E+03 | 1.26E+04 | 5.40E+02 | 2.57E+05 | 1.85E+04 | 1.77E+03 | 2.66E+04 | 1.19E+03 |
| 30 | 8.49E+05 | 3.58E+05 | 2.61E+04 | 2.58E+03 | 1.60E+02 | 2.01E+04 | 7.09E+03 | 9.80E+03 | 3.27E+04 | 3.40E+02 |
| 35 | 2.41E+06 | 8.72E+04 | 1.55E+05 | 5.43E+02 | 1.35E+02 | 5.22E+05 | 1.22E+05 | 4.57E+03 | 6.03E+03 | 1.41E+02 |

## *Phb. leiognathi* viability varied with GMP concentrations

MTT assays were performed to evaluate the effects of GMPs on the cell viability of both strains under different experimental conditions. Then, the ODs at 560 nm were measured and the mean OD_560_ are presented in Tables S5 and S6.

**Table S5.** The ODs at 560 nm and the mean ODs of *Phb. leiognathi* strains after 3.1 days GMP exposure at 30°C and 22°C.

| Temperature: 30°C | | | | | | | | | | |
| --- | --- | --- | --- | --- | --- | --- | --- | --- | --- | --- |
| Well | LB_01 | | | | | LB_09 | | | | |
|  | Control | GMP concentrations (w/v per mL) | | | | Control | GMP concentrations (w/v per mL) | | | |
|  |  | 0.25% | 0.5% | 1% | 2% |  | 0.25% | 0.5% | 1% | 2% |
| 1 | 0.251 | 0.239 | 0.273 | 0.420 | 0.387 | 0.264 | 0.256 | 0.210 | 0.214 | 0.271 |
| 2 | 0.242 | 0.391 | 0.280 | 0.497 | 0.338 | 0.342 | 0.210 | 0.248 | 0.270 | 0.295 |
| 3 | 0.257 | 0.246 | 0.274 | 0.480 | 0.372 | 0.302 | 0.325 | 0.245 | 0.236 | 0.273 |
| 4 | 0.282 | 0.282 | 0.282 | 0.390 | 0.337 | 0.228 | 0.349 | 0.244 | 0.265 | 0.258 |
| 5 | 0.239 | 0.301 | 0.250 | 0.358 | 0.327 | 0.298 | 0.384 | 0.268 | 0.228 | 0.244 |
| 6 | 0.362 | 0.301 | 0.358 | 0.412 | 0.400 | 0.307 | 0.403 | 0.214 | 0.331 | 0.230 |
| 7 | 0.374 | 0.277 | 0.334 | 0.360 | 0.287 | 0.340 | 0.408 | 0.330 | 0.241 | 0.266 |
| 8 | 0.368 | 0.400 | 0.274 | 0.392 | 0.301 | 0.261 | 0.205 | 0.252 | 0.296 | 0.268 |
| 9 | 0.352 | 0.375 | 0.273 | 0.399 | 0.345 | 0.253 | 0.267 | 0.321 | 0.241 | 0.242 |
| 10 | 0.344 | 0.289 | 0.355 | 0.463 | 0.412 | 0.207 | 0.254 | 0.275 | 0.373 | 0.275 |
| 11 | 0.367 | 0.355 | 0.364 | 0.393 | 0.433 | 0.335 | 0.264 | 0.228 | 0.339 | 0.232 |
| 12 | 0.235 | 0.387 | 0.337 | 0.425 | 0.356 | 0.288 | 0.323 | 0.253 | 0.239 | 0.243 |
| Mean | 0.306 | 0.320 | 0.305 | 0.416 | 0.358 | 0.285 | 0.304 | 0.257 | 0.273 | 0.258 |
| Median | 0.313 | 0.301 | 0.281 | 0.406 | 0.351 | 0.293 | 0.295 | 0.250 | 0.253 | 0.262 |
| SD | 0.059 | 0.058 | 0.041 | 0.044 | 0.044 | 0.044 | 0.072 | 0.037 | 0.051 | 0.020 |

**Table S5.** (Continuation)

| Temperature: 22°C | | | | | | | | | | |
| --- | --- | --- | --- | --- | --- | --- | --- | --- | --- | --- |
| Well | LB_01 | | | | | LB_09 | | | | |
|  | Control | GMP concentrations (w/v per mL) | | | | Control | GMP concentrations (w/v per mL) | | | |
|  |  | 0.25% | 0.5% | 1% | 2% |  | 0.25% | 0.5% | 1% | 2% |
| 1 | 0.260 | 0.203 | 0.239 | 0.265 | 0.205 | 0.269 | 0.189 | 0.222 | 0.206 | 0.226 |
| 2 | 0.305 | 0.174 | 0.205 | 0.191 | 0.208 | 0.272 | 0.138 | 0.224 | 0.232 | 0.203 |
| 3 | 0.325 | 0.216 | 0.183 | 0.206 | 0.195 | 0.248 | 0.146 | 0.252 | 0.140 | 0.272 |
| 4 | 0.295 | 0.225 | 0.225 | 0.214 | 0.177 | 0.255 | 0.152 | 0.124 | 0.247 | 0.291 |
| 5 | 0.295 | 0.234 | 0.200 | 0.128 | 0.131 | 0.185 | 0.122 | 0.153 | 0.278 | 0.200 |
| 6 | 0.279 | 0.176 | 0.181 | 0.122 | 0.173 | 0.232 | 0.112 | 0.164 | 0.168 | 0.186 |
| 7 | 0.259 | 0.200 | 0.222 | 0.222 | 0.117 | 0.203 | 0.249 | 0.136 | 0.216 | 0.238 |
| 8 | 0.273 | 0.209 | 0.258 | 0.142 | 0.120 | 0.265 | 0.125 | 0.227 | 0.256 | 0.240 |
| 9 | 0.263 | 0.179 | 0.232 | 0.160 | 0.147 | 0.291 | 0.258 | 0.153 | 0.256 | 0.207 |
| 10 | 0.246 | 0.229 | 0.252 | 0.161 | 0.140 | 0.285 | 0.227 | 0.147 | 0.232 | 0.295 |
| 11 | 0.333 | 0.177 | 0.287 | 0.166 | 0.128 | 0.209 | 0.123 | 0.138 | 0.156 | 0.220 |
| 12 | 0.354 | 0.204 | 0.247 | 0.145 | 0.108 | 0.182 | 0.182 | 0.227 | 0.143 | 0.256 |
| Mean | 0.291 | 0.202 | 0.228 | 0.177 | 0.154 | 0.241 | 0.169 | 0.181 | 0.211 | 0.236 |
| Median | 0.287 | 0.204 | 0.229 | 0.164 | 0.144 | 0.252 | 0.149 | 0.159 | 0.224 | 0.232 |
| SD | 0.034 | 0.022 | 0.032 | 0.043 | 0.036 | 0.038 | 0.052 | 0.046 | 0.048 | 0.036 |

**Table S6.** The ODs at 560 nm and the mean ODs of *Phb. leiognathi* strains after 7 days GMP exposure at 30°C and 22°C.

| Temperature: 30°C | | | | | | | | | | |
| --- | --- | --- | --- | --- | --- | --- | --- | --- | --- | --- |
| Well | LB_01 | | | | | LB_09 | | | | |
|  | Control | GMP concentrations (w/v per mL) | | | | Control | GMP concentrations (w/v per mL) | | | |
|  |  | 0.25% | 0.5% | 1% | 2% |  | 0.25% | 0.5% | 1% | 2% |
| 1 | 0.212 | 0.178 | 0.243 | 0.236 | 0.202 | 0.211 | 0.107 | 0.145 | 0.161 | 0.146 |
| 2 | 0.194 | 0.302 | 0.274 | 0.215 | 0.186 | 0.208 | 0.142 | 0.122 | 0.145 | 0.149 |
| 3 | 0.186 | 0.270 | 0.241 | 0.226 | 0.195 | 0.236 | 0.119 | 0.139 | 0.149 | 0.167 |
| 4 | 0.187 | 0.278 | 0.232 | 0.195 | 0.171 | 0.221 | 0.162 | 0.140 | 0.165 | 0.136 |
| 5 | 0.206 | 0.273 | 0.240 | 0.168 | 0.161 | 0.195 | 0.123 | 0.131 | 0.131 | 0.134 |
| 6 | 0.159 | 0.227 | 0.214 | 0.202 | 0.177 | 0.175 | 0.142 | 0.133 | 0.145 | 0.123 |
| 7 | 0.168 | 0.249 | 0.252 | 0.248 | 0.211 | 0.212 | 0.140 | 0.176 | 0.129 | 0.174 |
| 8 | 0.177 | 0.212 | 0.290 | 0.205 | 0.179 | 0.254 | 0.141 | 0.158 | 0.138 | 0.184 |
| 9 | 0.182 | 0.256 | 0.285 | 0.295 | 0.246 | 0.277 | 0.155 | 0.155 | 0.130 | 0.137 |
| 10 | 0.168 | 0.235 | 0.269 | 0.150 | 0.138 | 0.270 | 0.104 | 0.174 | 0.141 | 0.128 |
| 11 | 0.201 | 0.229 | 0.321 | 0.235 | 0.201 | 0.258 | 0.157 | 0.128 | 0.154 | 0.146 |
| 12 | 0.187 | 0.264 | 0.208 | 0.156 | 0.142 | 0.243 | 0.153 | 0.157 | 0.138 | 0.154 |
| Mean | 0.186 | 0.248 | 0.256 | 0.211 | 0.184 | 0.230 | 0.137 | 0.147 | 0.144 | 0.148 |
| Median | 0.187 | 0.253 | 0.248 | 0.210 | 0.183 | 0.229 | 0.142 | 0.143 | 0.143 | 0.146 |
| SD | 0.016 | 0.034 | 0.033 | 0.041 | 0.030 | 0.031 | 0.020 | 0.018 | 0.012 | 0.019 |

**Table S6.** (Continuation)

| Temperature: 22°C | | | | | | | | | | |
| --- | --- | --- | --- | --- | --- | --- | --- | --- | --- | --- |
| Well | LB_01 | | | | | LB_09 | | | | |
|  | Control | GMP concentrations (w/v per mL) | | | | Control | GMP concentrations (w/v per mL) | | | |
|  |  | 0.25% | 0.5% | 1% | 2% |  | 0.25% | 0.5% | 1% | 2% |
| 1 | 0.321 | 0.242 | 0.274 | 0.263 | 0.222 | 0.256 | 0.223 | 0.232 | 0.243 | 0.257 |
| 2 | 0.298 | 0.260 | 0.226 | 0.261 | 0.208 | 0.205 | 0.238 | 0.189 | 0.272 | 0.183 |
| 3 | 0.284 | 0.281 | 0.230 | 0.232 | 0.180 | 0.250 | 0.175 | 0.265 | 0.287 | 0.240 |
| 4 | 0.290 | 0.330 | 0.291 | 0.206 | 0.213 | 0.182 | 0.191 | 0.243 | 0.233 | 0.194 |
| 5 | 0.303 | 0.288 | 0.217 | 0.228 | 0.245 | 0.204 | 0.218 | 0.192 | 0.211 | 0.251 |
| 6 | 0.198 | 0.251 | 0.206 | 0.184 | 0.220 | 0.241 | 0.199 | 0.207 | 0.229 | 0.229 |
| 7 | 0.200 | 0.249 | 0.281 | 0.267 | 0.262 | 0.242 | 0.168 | 0.209 | 0.225 | 0.241 |
| 8 | 0.245 | 0.320 | 0.262 | 0.293 | 0.225 | 0.305 | 0.194 | 0.250 | 0.243 | 0.227 |
| 9 | 0.246 | 0.335 | 0.306 | 0.312 | 0.196 | 0.204 | 0.212 | 0.230 | 0.248 | 0.183 |
| 10 | 0.256 | 0.284 | 0.234 | 0.244 | 0.230 | 0.316 | 0.210 | 0.218 | 0.208 | 0.200 |
| 11 | 0.319 | 0.344 | 0.300 | 0.206 | 0.199 | 0.228 | 0.192 | 0.219 | 0.248 | 0.181 |
| 12 | 0.289 | 0.309 | 0.320 | 0.250 | 0.259 | 0.344 | 0.179 | 0.223 | 0.210 | 0.197 |
| Mean | 0.271 | 0.291 | 0.262 | 0.246 | 0.222 | 0.248 | 0.200 | 0.223 | 0.238 | 0.215 |
| Median | 0.287 | 0.286 | 0.268 | 0.247 | 0.221 | 0.242 | 0.197 | 0.221 | 0.238 | 0.214 |
| SD | 0.042 | 0.036 | 0.039 | 0.037 | 0.025 | 0.050 | 0.021 | 0.023 | 0.024 | 0.028 |

## Effects of GMPs in biofilm formation of *Phb. leiognathi* strains

The biofilm formation of the two strains was assessed by crystal violet biofilm formation assays following GMP exposure. The biofilms were quantified and expressed as SBF, which were presented in Tables S7 and S8.

**Table S7.** SBF of *Phb.leiognathi* strains after 3.1-day GMP exposure at 30°C and 22°C.

| Temperature: 30°C | | | | | | | | | | |
| --- | --- | --- | --- | --- | --- | --- | --- | --- | --- | --- |
| Well | LB_01 | | | | | LB_09 | | | | |
|  | Control | GMP concentrations (w/v per mL) | | | | Control | GMP concentrations (w/v per mL) | | | |
|  |  | 0.25% | 0.5% | 1% | 2% |  | 0.25% | 0.5% | 1% | 2% |
| 1 | 0.160 | 0.236 | 0.140 | 0.229 | 0.226 | 0.080 | 0.155 | 0.240 | 0.320 | 0.433 |
| 2 | 0.166 | 0.214 | 0.141 | 0.256 | 0.214 | 0.092 | 0.164 | 0.249 | 0.271 | 0.514 |
| 3 | 0.113 | 0.268 | 0.125 | 0.234 | 0.219 | 0.081 | 0.121 | 0.187 | 0.305 | 0.415 |
| 4 | 0.165 | 0.375 | 0.151 | 0.300 | 0.218 | 0.094 | 0.149 | 0.215 | 0.309 | 0.345 |
| 5 | 0.145 | 0.369 | 0.142 | 0.219 | 0.312 | 0.131 | 0.138 | 0.184 | 0.261 | 0.391 |
| 6 | 0.120 | 0.413 | 0.120 | 0.271 | 0.202 | 0.086 | 0.136 | 0.240 | 0.287 | 0.422 |
| 7 | 0.113 | 0.341 | 0.183 | 0.312 | 0.260 | 0.176 | 0.126 | 0.189 | 0.293 | 0.399 |
| 8 | 0.137 | 0.230 | 0.154 | 0.273 | 0.309 | 0.147 | 0.138 | 0.173 | 0.309 | 0.392 |
| 9 | 0.208 | 0.243 | 0.179 | 0.253 | 0.258 | 0.145 | 0.201 | 0.172 | 0.276 | 0.376 |
| Mean | 0.147 | 0.299 | 0.148 | 0.261 | 0.246 | 0.115 | 0.148 | 0.205 | 0.292 | 0.410 |
| Median | 0.145 | 0.268 | 0.142 | 0.256 | 0.226 | 0.094 | 0.138 | 0.189 | 0.293 | 0.399 |
| SD | 0.031 | 0.075 | 0.021 | 0.032 | 0.041 | 0.036 | 0.024 | 0.031 | 0.020 | 0.047 |

**Table S7.** (Continuation)

| Temperature: 22°C | | | | | | | | | | |
| --- | --- | --- | --- | --- | --- | --- | --- | --- | --- | --- |
| Well | LB_01 | | | | | LB_09 | | | | |
|  | Control | GMP concentrations (w/v per mL) | | | | Control | GMP concentrations (w/v per mL) | | | |
|  |  | 0.25% | 0.5% | 1% | 2% |  | 0.25% | 0.5% | 1% | 2% |
| 1 | 0.105 | 0.279 | 0.086 | 0.411 | 0.301 | 0.224 | 0.258 | 0.188 | 0.287 | 0.351 |
| 2 | 0.100 | 0.285 | 0.045 | 0.354 | 0.305 | 0.174 | 0.271 | 0.190 | 0.354 | 0.363 |
| 3 | 0.103 | 0.260 | 0.103 | 0.408 | 0.336 | 0.278 | 0.294 | 0.189 | 0.324 | 0.376 |
| 4 | 0.099 | 0.227 | 0.062 | 0.399 | 0.216 | 0.250 | 0.308 | 0.166 | 0.269 | 0.353 |
| 5 | 0.150 | 0.194 | 0.126 | 0.288 | 0.201 | 0.213 | 0.342 | 0.169 | 0.240 | 0.313 |
| 6 | 0.141 | 0.220 | 0.072 | 0.313 | 0.219 | 0.137 | 0.351 | 0.135 | 0.311 | 0.332 |
| 7 | 0.149 | 0.248 | 0.109 | 0.323 | 0.236 | 0.191 | 0.289 | 0.216 | 0.225 | 0.355 |
| 8 | 0.146 | 0.214 | 0.095 | 0.314 | 0.204 | 0.202 | 0.318 | 0.185 | 0.293 | 0.351 |
| 9 | 0.107 | 0.182 | 0.085 | 0.291 | 0.186 | 0.191 | 0.356 | 0.219 | 0.216 | 0.311 |
| Mean | 0.122 | 0.234 | 0.087 | 0.345 | 0.245 | 0.202 | 0.310 | 0.184 | 0.280 | 0.345 |
| Median | 0.107 | 0.227 | 0.086 | 0.323 | 0.219 | 0.191 | 0.308 | 0.188 | 0.287 | 0.351 |
| SD | 0.023 | 0.036 | 0.025 | 0.050 | 0.054 | 0.044 | 0.035 | 0.026 | 0.047 | 0.022 |

**Table S8.** SBF of *Phb.leiognathi* strains after 7-day GMP exposure at 30°C and 22°C.

| Temperature: 30°C | | | | | | | | | | |
| --- | --- | --- | --- | --- | --- | --- | --- | --- | --- | --- |
| Well | LB_01 | | | | | LB_09 | | | | |
|  | Control | GMP concentrations (w/v per mL) | | | | Control | GMP concentrations (w/v per mL) | | | |
|  |  | 0.25% | 0.5% | 1% | 2% |  | 0.25% | 0.5% | 1% | 2% |
| 1 | 0.354 | 0.193 | 0.275 | 0.252 | 0.370 | 0.183 | 0.216 | 0.199 | 0.270 | 0.357 |
| 2 | 0.371 | 0.153 | 0.140 | 0.186 | 0.232 | 0.260 | 0.134 | 0.278 | 0.218 | 0.420 |
| 3 | 0.351 | 0.099 | 0.181 | 0.204 | 0.263 | 0.205 | 0.115 | 0.262 | 0.228 | 0.344 |
| 4 | 0.277 | 0.172 | 0.161 | 0.206 | 0.205 | 0.147 | 0.225 | 0.202 | 0.240 | 0.340 |
| 5 | 0.311 | 0.121 | 0.229 | 0.273 | 0.310 | 0.100 | 0.197 | 0.222 | 0.263 | 0.333 |
| 6 | 0.318 | 0.136 | 0.222 | 0.190 | 0.271 | 0.193 | 0.225 | 0.231 | 0.312 | 0.395 |
| 7 | 0.296 | 0.133 | 0.171 | 0.216 | 0.226 | 0.165 | 0.174 | 0.281 | 0.279 | 0.389 |
| 8 | 0.293 | 0.133 | 0.226 | 0.218 | 0.258 | 0.206 | 0.206 | 0.292 | 0.257 | 0.345 |
| 9 | 0.296 | 0.188 | 0.168 | 0.244 | 0.369 | 0.222 | 0.132 | 0.223 | 0.244 | 0.345 |
| Mean | 0.319 | 0.148 | 0.197 | 0.221 | 0.278 | 0.187 | 0.180 | 0.243 | 0.257 | 0.363 |
| Median | 0.311 | 0.136 | 0.181 | 0.216 | 0.263 | 0.193 | 0.197 | 0.231 | 0.257 | 0.345 |
| SD | 0.033 | 0.032 | 0.043 | 0.029 | 0.060 | 0.046 | 0.043 | 0.035 | 0.029 | 0.030 |

**Table S8.** (Continuation)

| Temperature: 22°C | | | | | | | | | | |
| --- | --- | --- | --- | --- | --- | --- | --- | --- | --- | --- |
| Well | LB_01 | | | | | LB_09 | | | | |
|  | Control | GMP concentrations (w/v per mL) | | | | Control | GMP concentrations (w/v per mL) | | | |
|  |  | 0.25% | 0.5% | 1% | 2% |  | 0.25% | 0.5% | 1% | 2% |
| 1 | 0.197 | 0.243 | 0.241 | 0.358 | 0.378 | 0.357 | 0.319 | 0.182 | 0.305 | 0.259 |
| 2 | 0.256 | 0.270 | 0.302 | 0.403 | 0.365 | 0.350 | 0.225 | 0.172 | 0.268 | 0.237 |
| 3 | 0.242 | 0.253 | 0.297 | 0.404 | 0.420 | 0.318 | 0.192 | 0.225 | 0.258 | 0.234 |
| 4 | 0.234 | 0.214 | 0.298 | 0.394 | 0.359 | 0.304 | 0.134 | 0.218 | 0.289 | 0.245 |
| 5 | 0.231 | 0.168 | 0.266 | 0.440 | 0.326 | 0.364 | 0.177 | 0.245 | 0.247 | 0.320 |
| 6 | 0.268 | 0.187 | 0.255 | 0.457 | 0.356 | 0.329 | 0.194 | 0.229 | 0.218 | 0.265 |
| 7 | 0.254 | 0.171 | 0.282 | 0.402 | 0.364 | 0.315 | 0.243 | 0.198 | 0.290 | 0.315 |
| 8 | 0.135 | 0.208 | 0.278 | 0.443 | 0.379 | 0.244 | 0.122 | 0.214 | 0.336 | 0.330 |
| 9 | 0.230 | 0.202 | 0.278 | 0.369 | 0.361 | 0.285 | 0.133 | 0.194 | 0.340 | 0.289 |
| Mean | 0.227 | 0.213 | 0.277 | 0.408 | 0.368 | 0.318 | 0.193 | 0.209 | 0.283 | 0.277 |
| Median | 0.234 | 0.208 | 0.278 | 0.403 | 0.364 | 0.318 | 0.192 | 0.214 | 0.289 | 0.265 |
| SD | 0.040 | 0.036 | 0.021 | 0.033 | 0.025 | 0.038 | 0.063 | 0.024 | 0.040 | 0.037 |

# Supplementary Figure

## Size Distribution of GMPs

The size of GMPs was measured using ImageJ software. Before measuring the GMPs, a known scale bar (0.001 mm) was used for calibration. Then, pre-captured micrographs of GMPs were obtained. A total of 1,292 particles were measured, and the Feret diameter (the maximum caliper length) of each particle were recorded and expressed in micron (µm). The size distribution was determined as showed in **Figure S1**.


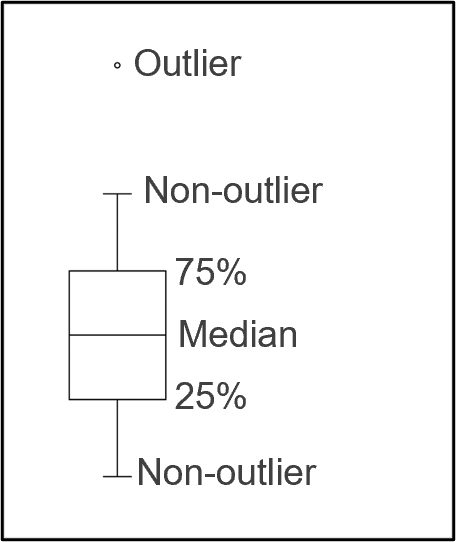


**B**

**A**


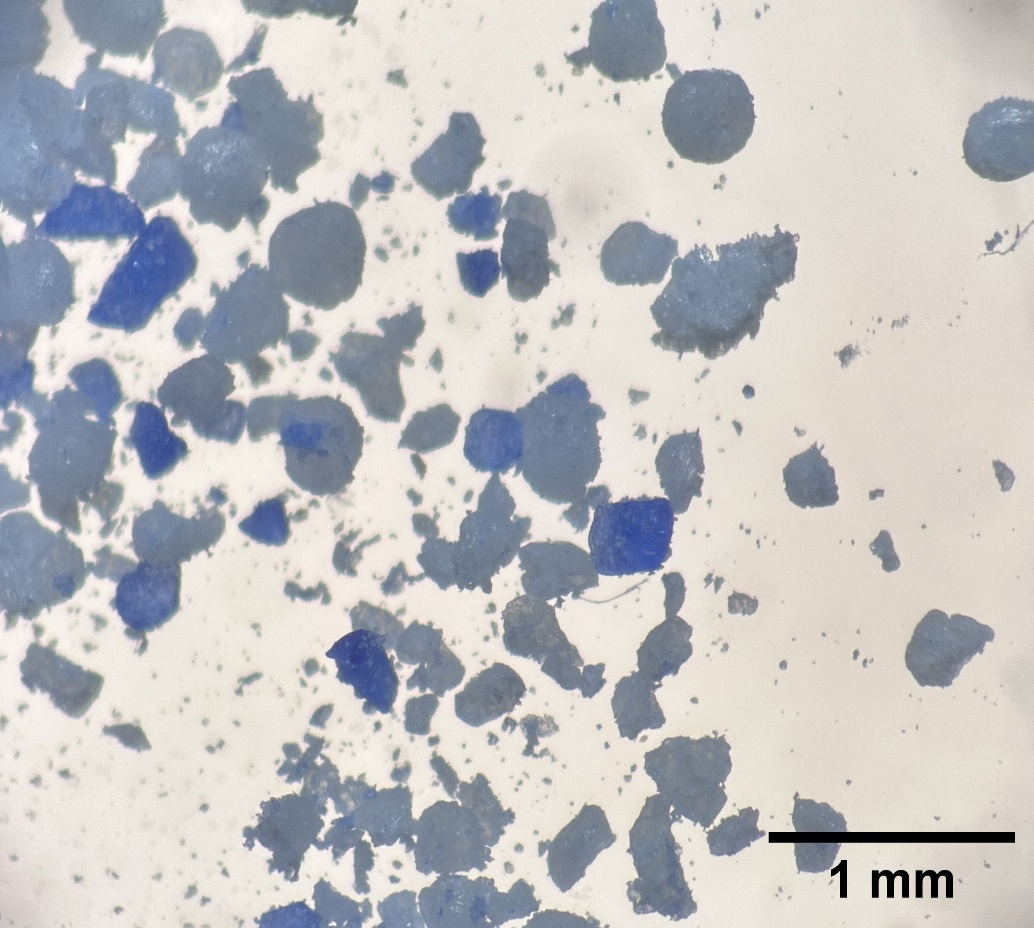

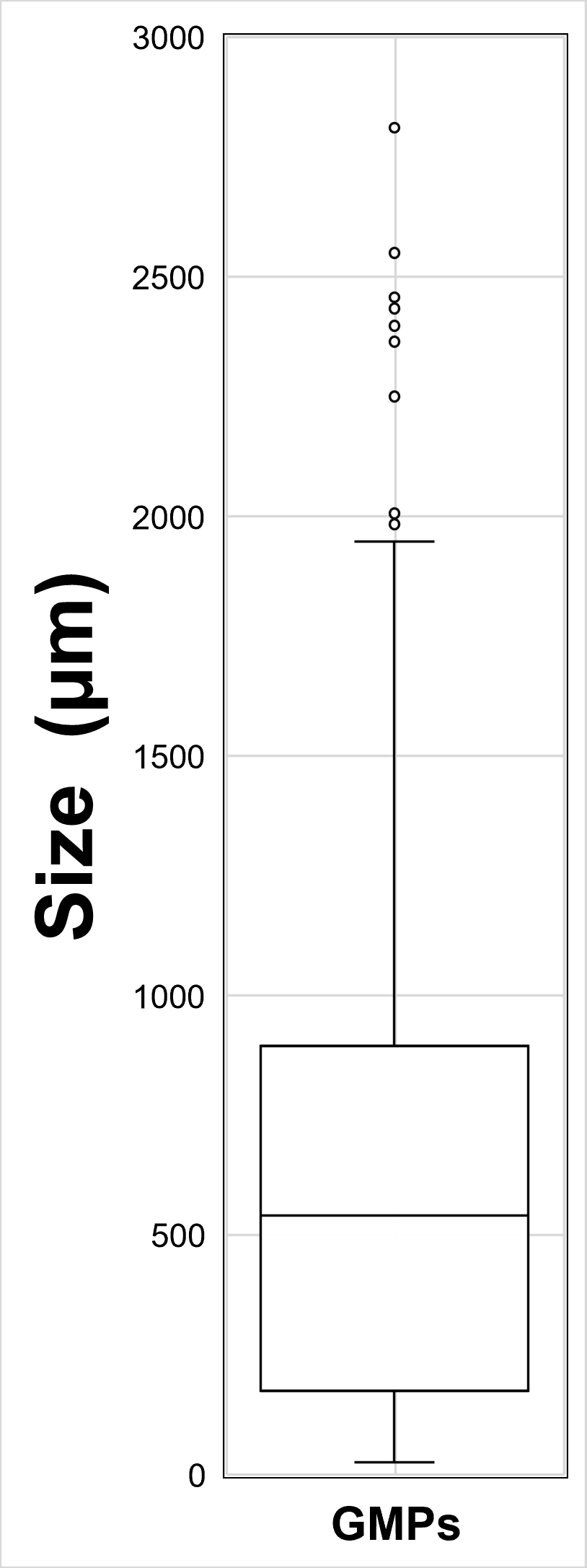


**Figure S1.** Size distribution of GMPs. (**A**) The mean size of GMPs was 633.37 µm, while the median size was 540.85 µm. The first quartile (Q1, 25^th^ percentile) was 175.98 µm, and the third quartile (Q3, 75^th^ percentile) was 894.13 µm. The largest GMP measured was 2.81 mm, and the smallest was 25.5 µm. (**B**) Micrograph of GMPs as viewed under the stereomicroscope.
